# Protecting Galápagos’ marine ecosystems: Biosecurity and network design against invasive species from tourist vessels

**DOI:** 10.1016/j.isci.2026.115486

**Published:** 2026-03-26

**Authors:** Marnie L. Campbell, Chi T.U. Le, Chad L. Hewitt

**Affiliations:** 1 School of Science, Edith Cowan University, 270 Joondalup Drive, Joondalup WA 6027, Australia; 2 School of Life and Environmental Science, Deakin University, Waurn Ponds VIC 3216, Australia; 3 Centre for One Biosecurity Research, Analysis and Synthesis, Lincoln University, PO Box 85084, Lincoln, Christchurch 7647, New Zealand

**Keywords:** Marine organism, Environmental science, Nature conservation

## Abstract

The modern world is characterized by high connectivity, enhancing economic productivity, and global communication, but outpacing our ability to manage its consequences. This connectivity increases the risk of pathogen, pest, and invasive species transmission. In the Galápagos islands, Ecuador, tourist vessel networks operate with limited restrictions and connect high conservation value locations, boosting tourism and the economy. These vessels frequently travel between multiple islands, leading to moderate connectivity and high vessel promiscuity. To prevent the spread of invasive species via these vessels, we propose a biosecurity system that combines social network tools with risk management frameworks that balances environmental protection with economic values. Our research suggests that leveraging environmental mismatches between island pairs and creating small-world networks can provide a functional and cost-effective biosecurity solution. These findings have global relevance for managing the spread and impact of invasive species worldwide.

## Highlights

• Small tourist vessels risk spreading invasive species across the Galapagos islands
• Network analysis enables cost-effective biosecurity planning and management
• Environmental matching can be integrated into the network to inform management
• Zoning based on network communities can minimize the impacts of biosecurity actions

## Graphical abstract

**Figure:**
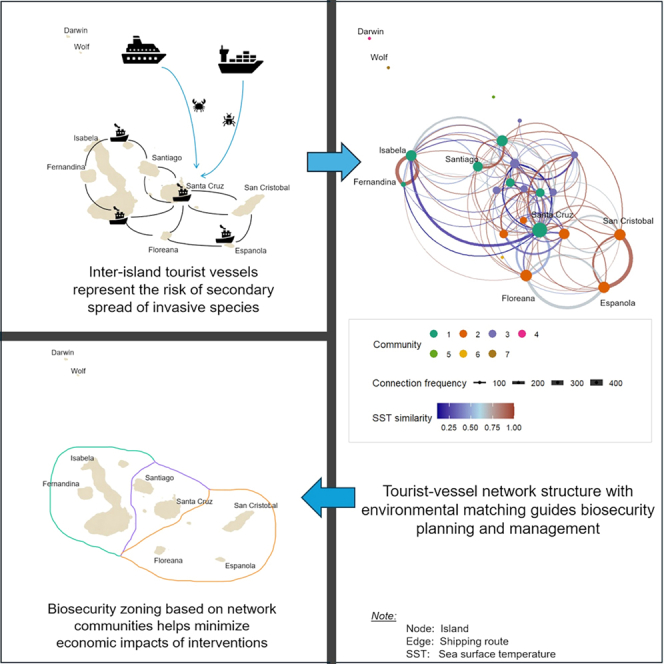

## Introduction

We live in a world where we are hyperconnected (*sensu* Quan-Haase and Wellman 2005) through rapid air, land, and maritime transport. Our global connectivity brings both economic and social benefits^1^ and real environmental and social costs (e.g., exposure to pandemics^2^^,^^3^^,^^4^^,^^5^; pollution^6^^,^^7^^,^^8^). Specifically, connectivity increases the vulnerability to the transfer and establishment of novel biota in ecosystems. For example, the increased connections between ports and bioregions result in greater exposure to risks such as pandemics,^9^^,^^10^^,^^11^ financial crises,^4^ and non-indigenous marine species (NIMS).^12^^,^^13^^,^^14^^,^^15^^,^^16^^,^^17^ Furthermore, port exposure to promiscuous vessels (i.e., vessels that visit many ports) increases the risk of infection by NIMS.^13^^,^^18^^,^^19^^,^^20^ Vessels trading with “infected” regions are more likely to become infected and act as transfer agents.^12^^,^^13^

Often, the management of introduced species, pathogens, pests, or diseases focuses on stopping the incursion event from occurring,^21^^,^^22^^,^^23^ rather than restrictions on connectivity as mitigation options.^24^ Marine biosecurity could benefit from incorporating lessons from pandemic health management and risk messaging.^25^^,^^26^ The primary focus of pandemic health management response is halting the spread of the disease and controlling messaging to calm citizens e.g.,^3^ rather than addressing the mechanism of arrival and spread.

Our ability to react to NIMS incursions relies on biosecurity planning,^27^^,^^28^ including risk consequence assessment (what could be affected), risk planning (how to manage the impacts), risk communication,^27^ and identifying critical decisions.^25^^,^^26^^,^^29^^,^^30^ Biosecurity managers often create different risk scenarios to test pro-active and reactive strategies. There are various management mechanisms available to control the entry risks, including policies and border control mechanisms e.g., entry requirements, import controls, risk communication, screening tools to aid detection, international instruments, inspection and surveillance regimes, fines; e.g.,.^20^^,^^21^ Quarantine, eradication, or management occurs once a NIMS has arrived and established.^27^^,^^30^

Several factors, beyond the influence of biosecurity managers, contribute to the enhanced risk of NIMS arrivals and establishments, including vessel size and wettable surface area,^31^^,^^32^ vessel maintenance regimes,^19^^,^^33^ and vessel exposure duration in the previous ports of call.^13^^,^^31^ These factors may enhance prediction of dispersal risks e.g.,^34^ providing screening opportunities and filters, but rarely provide on ground options that biosecurity managers can readily influence. The use of “environmental matching” between donor and recipient locations, such as temperature and salinity differences, can be used to highlight relative changes in survival risk.^20^^,^^27^^,^^35^^,^^36^

**Figure 1:**
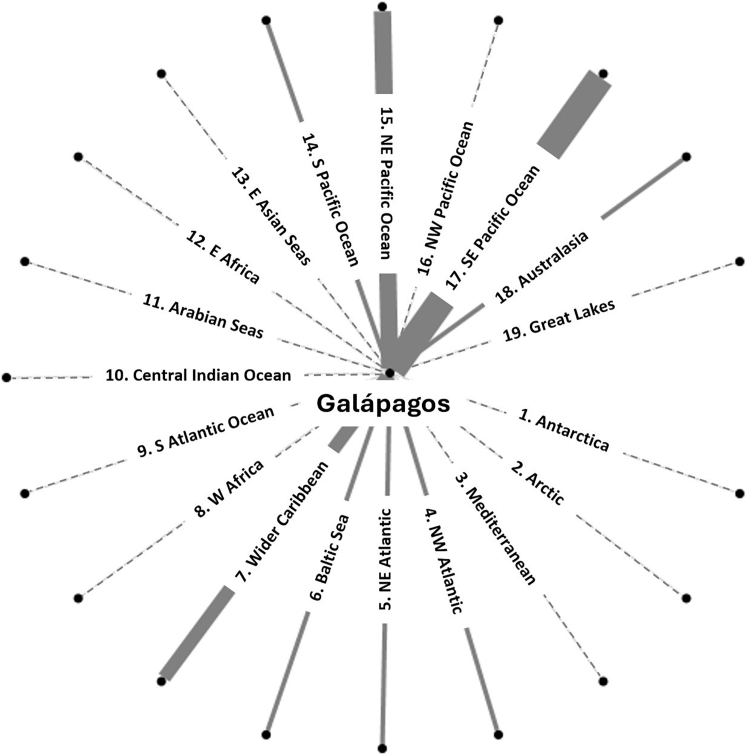
International trading connections with the Galápagos islands to the different IUCN bioregions (referring to the 18 IUCN bioregions) Thickness of a line (edges) represents the strength of connection between the Galapagos and an IUCN bioregion. A thicker line represents more connection via international maritime vessel movement. A dashed line represents no maritime vessel connection. An outer point represents a “node” (port or maritime location) within the IUCN bioregion that is connected to the Galapagos.

In this study, we use the Galápagos islands (Ecuador) as an example to investigate how anthropogenic connectivity could be used to create simple and smart biosecurity management options. The Galápagos islands are poorly connected by currents and wind see,^37^ however, intensely connected through vessel shipping movements, including recreational, tourism, fishing, military, and commercial vessels (Figure 1). Maritime traffic facilitates the introduction and spread of NIMS by transporting marine organisms attached to vessel hulls (biofouling species) or contained within ballast water and other water-holding compartments across regions.^38^^,^^39^^,^^40^ Introduced species (both terrestrial and marine) are a significant concern in the region.^40^^,^^41^^,^^42^^,^^43^^,^^44^^,^^45^ The Galápagos islands rely on tourism and fisheries,^46^ which benefit from effective park management^47^ and are potentially impacted by introduced species.^40^^,^^48^ By using the Galápagos islands as an example, we aim to highlight the importance of considering connectivity in biosecurity planning and management. Specifically, we seek to understand the network of shipping connections, the linkage “risk” based on temperature differences, and their potential impact on the spread of NIMS within the Galápagos islands.

To do that, we examined small-vessels involved in ferrying people between islands (interisland ferries) and tourist destinations, and small-vessels associated with tourism activities on daytrips (island hopping vessels). These vessels collectively represented approximately 13.5% of all vessels visiting and operating within the archipelago (2015 data,^49^). Although they account for a relatively small proportion, their frequent interisland movement heightens their risk as key vectors for NIMS spread. Excluded are recreational (including recreational fishing) and live aboard tourism vessels due to data unavailability. Table S1 provides an overview of the tourism activities that occur across the region, where the tourism small-vessels engage.

## Results

### Domestic tourist small-vessel traffic as a weighted network

The Galápagos Islands, as a weighted network with the frequency of trips between each island (node) pair serving as link/connection weights, formed a small-world network structure (S = 1.75). The network’s undirected average shortest path length (L_g_ = 0.013) was not significantly different from that of a random network (*p* = 0.576), while its global clustering coefficient (C_g_ = 0.598) was significantly greater than that of a random network (*p* < 0.001).

During our study time frame, the archipelago had a total of 12,433 vessel trips connecting 19 out of 23 islands within the region, with 55.3% of the trips being reciprocal between island pairs, and the rest being one-way trips. There were 28% ± 5.7% (SE) connections between the Harris SST zones, and 55% ± 18.3% (SE) within the zones (Table S2). Santa Cruz was the most connected island, which sent traffic to 16 islands and received traffic from 14 islands within the archipelago, with traffic intensity (strength) of 1963 and 1810, respectively. This island also had the greatest betweenness (how often Santa Cruz sits on the shortest path connecting two other islands) and out-closeness (how easily infections spread to other islands). Rankings of individual islands within the archipelago based on four network metrics, including out-degree (total number of connections to other islands), out-strength (total weighted connections to other islands), betweenness, and out-closeness, are presented in Table 1. The network metrics for each island are illustrated in Table S3. The Leiden clustering indicated that the archipelago formed seven communities, four of which included one isolated island each (i.e., Darwin, Pinta, Pinzon, and Wolf). The network had a modest modularity (Q = 0.286), which was not greater than expected by chance under a degree-preserving randomization (*z* = 1.562, *p* = 0.118, two-sided, 1,000 permutations). This suggests the whole network was a locally close-knit structure, but not partitioned into strongly distinct communities. Hence, the detected communities were treated as illustrative only.

**Table 1:** Ranking of individual Galápagos islands by different network indices (i.e., different network metrics)

Ranking	Out-degree	Out-strength	Betweenness	Out-closeness
1	Santa Cruz	Santa Cruz	Santa Cruz	Santa Cruz
2	Santiago	Isabela	Genovesa	Floreana
3	Bartolome	Santiago	Floreana	Bartolome
4	Genovesa	Floreana	Isabela	Santiago
5	Floreana	Espanola	Bartolome	Espanola
6	Isabela	San Cristobal	Santiago	Genovesa
7	North Seymour	Bartolome	Santa Fe	San Cristobal
8	Espanola	Genovesa	Espanola	Rabida
9	San Cristobal	Santa Fe	San Cristobal	North Seymour
10	Rabida	Fernandina	North Seymour	Sombrero Chino
11	Sombrero Chino	Rabida	Rabida	Isabela
12	Mosquera	Sombrero Chino	South Plaza	Santa Fe
13	Santa Fe	North Seymour	Daphne Major	Daphne Major
14	South Plaza	South Plaza	Baltra	Plazas
15	Baltra	Baltra	Plazas	Fernandina
16	Plazas	Plazas	Fernandina	South Plaza
17	Fernandina	Daphne Major	Sombrero Chino	Baltra
18	Daphne Major	Mosquera	Mosquera	Mosquera
19	Marchena	Marchena	Marchena	Marchena

Out-degree - total number of connections to other islands; Out-strength - total weighted connections to other islands; Betweenness - how often an island lies on the shortest paths between other vertices; Out-closeness - how easily infections spread to other islands.

Note: Disconnected islands, including Darwin, Pinta, Pinzon, and Wolf, were excluded.

**Figure 2:**
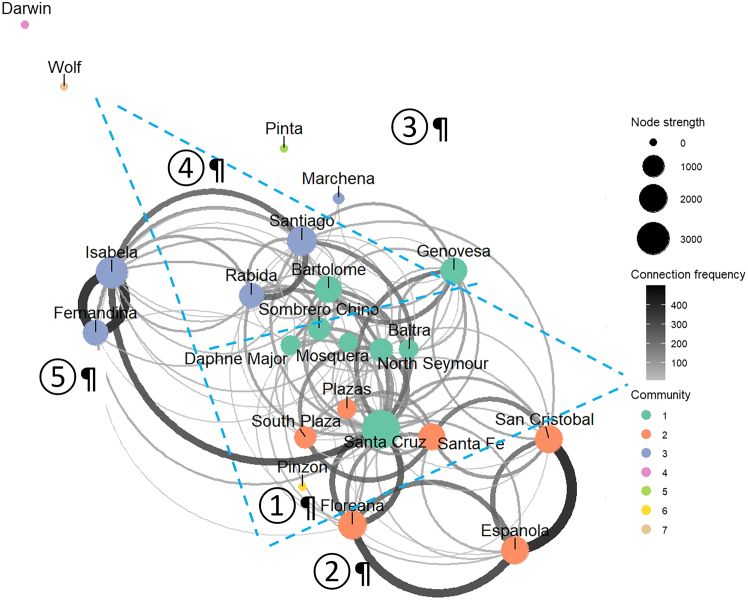
The Galápagos islands directed weighted network with the color-coded seven communities based on the Leiden method The blue dashed lines indicate the boundaries of the Harris SST zones, which are labeled by the numbers within circles.

The active vessel connections formed three (weak) communities (excluding four disconnected islands) (Table 2). Community 1 consisted of eight islands, including Baltra, Bartolome, Daphne Major, Genovesa, Mosquera, North Seymour, Santa Cruz, and Sombrero Chino. This community had the largest internal flow, inflow, and outflow traffic, as well as the largest number of bridges to the other communities. Community 2 with six islands (Espanola, Floreana, Plazas, San Cristobal, Santa Fe, and South Plaza) came second in traffic, but exhibited the highest exposure (highest in-closeness). In contrast, community 3, consisting of five islands (Fernandina, Isabela, Marchena, Rabida, and Santiago), while having the lowest traffic, had the second-highest number of bridges, and the highest export pressure (i.e., largest out-closeness). Figure 2 illustrates the communities and connections within the network.

**Table 2:** Galápagos islands community vulnerability metrics

Comm	Size	No of bridges	Internal flow	Total inflow	Total outflow	In-closeness	Out-closeness	Average between--ness
1	8	55	2887	2152	2203	67.49	64.66	6.75
2	6	33	2776	1195	1129	107.93	113.9	4.67
3	5	34	2156	1267	1282	59.02	125.26	2.4

Note: membership: 1 – Baltra, Bartolome, Daphne Major, Genovesa, Mosquera, North Seymour, Santa Cruz, and Sombrero Chino; 2 – Espanola, Floreana, Plazas, San Cristobal, Santa Fe, and South Plaza; 3 – Fernandina, Isabela, Marchena, Rabida, and Santiago. Disconnected islands were excluded, including Darwin, Pinta, Pinzon, and Wolf.

### Domestic tourist small-vessel traffic—A network with SST-integrated composite weights

The Kruskal-Wallis test indicated a statistically significant omnibus difference in the SST similarity across the 12 investigated months from February 2015 to January 2016 (χ^2^ = 209.34, df = 11, *p* < 0.001). The pairwise Dunn’s tests showed that April, June, and July had the highest SST similarity (median = 0.84), while March, August, and September had the lowest SST similarity (median = 0.49) (Figure S1 and Table S4). Mean island SST differences for all pairwise island combinations by month are presented in matrix form in Figure S2, and as a network visualization in Figure S3. For illustrative purposes, we present the network statistics for April as a representative of the highest SST similarity month (Table S5). Since the node degree did not change when SST similarity was considered, this metric is excluded from the results presented in Table 3.

**Table 3:** Ranking of the Galapagos islands’ invasion risk by different indices (i.e., different network metrics) for April with SST similarity incorporated

Ranking	Out-strength	Betweenness	Out-closeness
1	Santa Cruz (1)	Santa Cruz (1)	Santa Cruz (1)
2	Isabela (2)	Genovesa (2)	Santiago (4)
3	Santiago (3)	Santiago (6)	Rabida (8)
4	San Cristobal (6)	Isabela (4)	Isabela (11)
5	Espanola (5)	Santa Fe (7)	Genovesa (6)
6	Fernandina (10)	Bartolome (5)	Floreana (2)
7	Floreana (4)	San Cristobal (9)	San Cristobal (7)
8	Rabida (11)	Floreana (3)	Fernandina (15)
9	Genovesa (8)	Espanola (8)	Espanola (5)
10	Bartolome (7)	North Seymour (10)	Bartolome (3)
11	Santa Fe (9)	Rabida (11)	Santa Fe (12)
12	South Plaza (14)	Baltra (14)	Sombrero Chino (10)
13	Sombrero Chino (12)	South Plaza (12)	North Seymour (9)
14	Baltra (15)	Sombrero Chino (17)	South Plaza (16)
15	North Seymour (13)	Daphne Major (13)	Baltra (17)
16	Daphne Major (17)	Fernandina (16)	Plazas (14)
17	Plazas (16)	Plazas (15)	Daphne Major (13)
18	Mosquera (18)	Mosquera (18)	Marchena (18)
19	Marchena (19)	Marchena (19)	Mosquera (17)

Note: Disconnected islands, including Darwin, Pinta, Pinzon, and Wolf, were excluded. Numbers in parentheses = rankings from Table 1.

**Figure 3:**
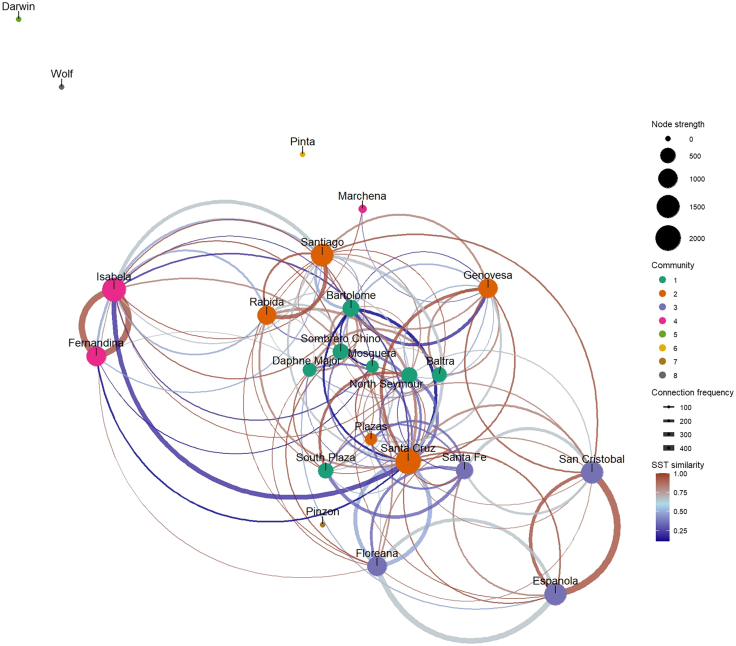
The Galápagos islands directed weighted network with April SST similarity incorporated The communities, detected based on the Leiden method, were color-coded. Edge color indicates similarity in SST between a pair of nodes. Edge thickness represents the frequency of trips between a pair of nodes. SST similarity of 0.6 (equivalent to an SST difference of 2°C) was chosen as the midpoint of the scale.

The incorporation of SST similarity into the network analysis altered edge weights (inter-node relationships) and node statistics, leading to changes in node ranking (Table 3) and community structure. Community structure was moderate (modularity Q = 0.34), and borderline greater than expected under a degree-preserving null model (*z* = 1.93, two-sided *p* = 0.053, 1,000 permutations). Four interconnected communities were formed in the network, with their vulnerability metrics illustrated in Table 4. Figure 3 shows the network with communities and SST similarity color-coded.

**Table 4:** Galápagos islands community vulnerability metrics with April SST similarity incorporated

Comm.	Size	No of bridges	Internal flow	Total inflow	Total outflow	In-closeness	Out-closeness	Average betweenness
1	7	49	914.25	872.96	708.39	22.95	26.88	7.29
2	5	55	1477.54	1281.47	1342.99	69.05	74.22	1.80
3	4	26	1581.68	784.93	652.62	133.36	125.86	1.50
4	3	20	947.49	295.74	531.10	44.56	315.63	0.33

Note: membership: 1 – Baltra, Bartolome, Daphne Major, Mosquera, North Seymour, Sombrero Chino, and South Plaza; 2 – Genovesa, Plazas, Rabida, Santa Cruz, and Santiago; 3 – Espanola, Floreana, San Cristobal, and Santa Fe; and 4 – Fernandina, Isabela, and Marchena. Disconnected islands were excluded, including Darwin, Pinta, Pinzon, and Wolf.

## Discussion

We use the Galápagos Islands as a case study to demonstrate how network analysis of tourist vessel connectivity can support biosecurity planning and management, and how environmental variables (e.g., SST) can be integrated into the network to inform management. Our findings suggest that the domestic tourist vessel traffic across the islands within the archipelago exhibited small-world characteristics,^50^ making it vulnerable to the spread of NIMS within the region once they are introduced. This demonstration represents a significant increased risk by controllable anthropogenic activity relative to natural dispersal by currents and wind.^37^ While marine biosecurity planning and management require consideration of the power of the islands in spreading species, taking into account the islands’ shipping values could improve the cost-effectiveness of the strategy. Our network analysis serves as a tool for these considerations.

During the study period, Santa Cruz was understandably confirmed as the primary local transport hub, showing the highest in- and out-degree, strength, betweenness, and out-closeness. As the main maritime link to mainland Ecuador, it had the greatest potential to be a primary site of NIMS infection that could then potentially spread out to its connected islands (secondary dispersal via tour vessels^51^). If a NIMS incursion occurs on this island, control efforts (e.g., limited navigation or quarantine) could disrupt intensive tourist links to neighboring islands, posing negative economic impacts on the region. This underscored the need for strict pre-arrival inspections before vessels enter Santa Cruz.

In contrast, four islands (Pinzon Island in Harris zone 1; and Darwin, Wolf, Pinta islands in Harris zone 3) remained unconnected to the tourist vessel traffic examined in our study. Darwin and Wolf islands are the two northernmost islands that were not represented in our scheduled tourist small-vessel data. The lack of representation of these islands might be due to their distance from the Santa Cruz hub (317 km and 278 km, respectively), which may reduce general tourist small-vessel interest and capability. We note that specialist tourist groups are more likely to charter a larger vessel (e.g., live aboard cruise vessels) to visit these locations, operating when capacity is met, rather than via a scheduled/timetabled calendar. Darwin and Wolf are known dive and research destinations,^52^^,^^53^ and frequented by live aboard cruise vessels withexpeditions lasting from 4 to 7 days, for example (e.g., https://www.voyagers.travel/galapagos/galapagos-islands-information/islands/galapagos-island-santa-cruz).

The ranking of the 19 connected islands (e.g., Tables 1 and 3) represents their power of spreading species, making it a useful tool for planning. For example, in 2016, the Ecuadorian government was asked by the World Heritage Committee (WHC/16/40.COM/19; https://whc.unesco.org/archive/2016/whc16-40com-19-en.pdf) to implement a proposal designating Baltra Island as the authorized point of entry for cargo from the mainland port of Guayaquil (https://whc.unesco.org/en/soc/3450). Establishment of a dedicated cargo facility at Baltra Island would move the primary entry transport hub (and its associated exposure to threats) away from Santa Cruz and align Baltra Island as the transport hub across all transport pathways (air flights, naval, and merchant maritime traffic). From a network perspective, Baltra was ranked low (14^th^ to 17^th^) across various indices (Tables 1 and 3), indicating a relatively lower risk of species spread via tourist vessels. Because Baltra Island has already experienced significant ecological degradation,^54^^,^^55^^,^^56^^,^^57^ concentrating merchant shipping and cargo operations there may help contain further impacts to a single site, thereby reducing the likelihood of exposing more pristine islands to risk. We note that the full transition of maritime shipping to Baltra has not yet occurred. This island currently serves as a hub for live aboard vessels that refuel at this location, and cargo vessels offload cargo on the northern side of Santa Cruz Island. The implementation of the proposed strategy would indeed shift the connectivity across the archipelago, potentially affecting tourist vessel routes and traffic patterns. Applying network structure would guide prioritization of efforts on high-risk islands while balancing their shipping values (Shi et al., 2024).

Community detection identified “neighborhoods” (i.e., communities of islands detected via network analysis) based on trip frequency (Figure 2) and environmental SST differences/similarity (Figure 3), focusing biosecurity attention on the most connected nodes in a community and key vessel connections (bridges) to other communities.^58^ Our findings indicate a moderate modularity of the directed weighted network, suggesting that intra-community connections are relatively weak, with substantial inter-community links (Tables 2 and 4). Incorporating SST into the network improved its modularity, implying stronger links within communities where SST similarity was high (Figure 3). With a moderate network modularity, biosecurity management at the community level should prioritize monitoring and control efforts on bridging nodes/edges between communities. The community vulnerability metrics serve as measurements of tourist shipping values (Table 2) and import and export (of species) risk at the community level (Table 4).

**Figure 4:**
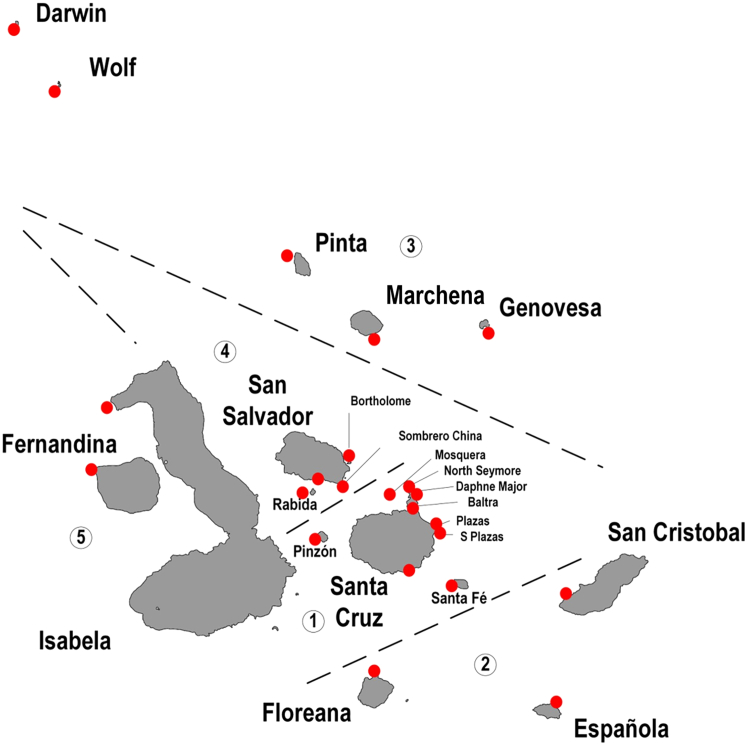
Galápagos Islands, with zones 1–5 (separated by dashed lines) based on sea surface temperature (SST) (Harris 1969), referred to as Harris SST zones. Red dots indicate SST sampling locations Base map Andrew Z. Colvin, CC BY-SA 4.0, via Wikimedia Commons.

The Galápagos Islands cover a broad range of environmental attributes, specifically SST^59^^,^^60^ These temperatures vary greatly between islands throughout the year (Figures 4, S2, and S3), with the greatest relative variation observed during the dry/cool season from September to November. As climate change continues to progress, these intra-annual differences may also shift. Paltán and colleagues (2021)^60^ reported an overall increase of 1.2°C in the SSTs from 2002 to 2018, with a larger increase in the northern islands (Harris zone 3) and a cooling trend in other regions (Harris zones 1, 2, 4, and 5). Since environmental matching (similarity between source and destination conditions) influences benthic (biofouling) species survival and establishment risks,^20^^,^^35^^,^^36^ adding SST similarity to the transport network highlights connections with elevated risk.

In our study, SST data were extracted at the island-scale resolution, and monitoring sites do not necessarily coincide with specific ports or anchoring locations, especially in large islands (e.g., Isabela). SST values, therefore, represent island-level general conditions rather than fine-scale environmental matching at ports. Incorporating finer-scale, port-specific environmental data would further enhance future biosecurity risk assessment.

We note that other environmental attributes, such as salinity, may also be useful in considering environmental matching. Salinity can directly impact species osmoregulatory processes, especially in larval stages, and influence thermal tolerance.^61^ Salinity in the Galápagos Islands varies between 33.8ppt and 34.9ppt driven by the influence of three current driven water masses, surface evaporation and low levels of freshwater input.^62^^,^^63^ Observed salinity variability in the region is primarily temporal rather than spatial, with differences across the archipelago small relative to seasonal variation.^64^ While species’ tolerances to salinity have been empirically evaluated for a relatively small number of species, e.g.,^13^ this variation is unlikely to create sufficient differentiation between individual islands to exceed benthic species’ tolerances.

We acknowledge that the composite weights for network connections, computed as the product of trip frequency and SST similarity, serve as a proxy product of propagule pressure and environmental matching, rather than a direct measure of realized species dispersal risk. Accordingly, we used these weights only to rank the nodes and edges by their relative importance within the network.

Since 2007, the interisland domestic vessel traffic has increased,^40^ with at least 116 daily cruise boat operators and 78 cruise ship providers based and operating in the islands.^65^ Small boat tours represented 12% of tourist service providers, and 37% of direct tourist jobs in the Galápagos.^65^ We identified a total of 34 unique domestic tourist vessels within this network, but we did not determine ownership/operators as this information was sparse. Among those tourist vessels, no vessel visits all 19 of the active islands; however, a moderate number of vessels visit between 13 and 17 islands, providing evidence of vessel promiscuity in the Galápagos islands. The spread of tourist vessel operators and visitor destinations, as noted by Burbano et al.,^65^ would help prevent monopolization in the domestic marine tourism sector (unlike what is seen in other island regions such as the Mediterranean;^8^).

Given that economic activity is largely limited to tourism, fisheries, and governance-dependent activities (e.g., conservation), and that the Galapagos National Park regulates the tourism vessel itineraries, we propose an additional biosecurity overlay that treats tourism small-vessels as vectors of NIMS to improve marine biosecurity. Specifically, we suggest (1) zoning routes into “neighborhoods” based on geography and SST similarity (e.g., Figure 3); (2) allowing operator diversification within neighborhoods to increase economic resilience; and (3) encouraging multi-vessel fleets distributed across neighborhoods to provide economic continuity e.g.,^66^ and buffer shocks when singular or multiple events occur (e.g., vessel breaking down, oil spill, or quarantine due to a NIMS incursion). Movements between neighborhoods would be conditioned under biosecurity standards. If a NIMS incursion occurs in a specific community, cross-neighborhood itineraries can be paused to prevent NIMS spread, while control and eradication efforts are underway. Operators can continue itineraries within the affected neighborhood, ensuring that economic impacts are minimized. Impacts on business operations would be ameliorated if tourist operators had vessels operating in separate neighborhoods without routine inter-neighborhood rotation.

We note that our focus is on addressing the challenge posed by non-indigenous marine species spread within the Galápagos Islands to ensure sustainability and support the continuation of industries such as fisheries and tourism. However, we are not political or economic analysts and hence our recommendations (such as the creation of neighborhoods with operator and vessel regulations) are based on scientific and environmental governance needs, which may not fully address the complex social-economic issues of the region. Other authors^67^^,^^68^^,^^69^ have explored these issues in greater depth.

Currently, San Cristobal is designated as the main port-of-entry to the archipelago for all vessels. This arrangement is likely to change vessel movement patterns, including tourist vessels’, and consequently, redistribute invasion risk within the archipelago. Periodically re-estimating the network with recent movement data would enable regulatory bodies to target surveillance and response at the highest-risk links and locations.

Cost-effective marine biosecurity in the Galápagos Islands should balance biosecurity and economic outcomes. Network analysis to integrate shipping activity and environmental matching allows managers to map where propagule pressure and environmental suitability are highest, rank routes and ports by relative importance, and target surveillance and interventions for greatest return. Embedding these insights in zoning and conditional cross-neighborhood movements can curb the spread of NIMS during incursions while sustaining tourism. This provides a transparent basis for prioritizing resources and coordinating rapid response to NIMS across the archipelago. However, its successful implementation requires the involvement of a wide range of stakeholders to create a co-managed system that works for the islands. This is achievable, as evidenced by the evolving marine biosecurity in the Galápagos islands.^34^^,^^70^

### Limitations of the study

It is noted that, in addition to small tourist boats covered in this study, private yachts, tourism live-aboard cruisers, fishing boats, and domestic patrol vessels are permitted to travel within the Galápagos archipelago, albeit under strict regulatory constraints, including authorization, designated ports of call, and operational zoning.^49^^,^^71^ Although these vessels may contribute to ship-induced secondary invasion within the islands, systematic data on their inter-island routes and movement frequency are either limited or unavailable. This data gap prevents their explicit inclusion in the shipping-based network analysis, and potentially results in an underestimation of local-scale invasion risk. However, the models developed herein provide a management tool that would readily apply to the other vessels.

## Resource availability

### Lead contact

Further information and requests for resources should be directed to and will be fulfilled by the lead contact, Marnie Campbell (marnie.campbell@ecu.edu.au).

### Materials availability

This study did not generate new unique reagents.

### Data and code availability

• The data reported in this study is available from the lead contact upon request.
• The R code used for the analysis in this research is provided in section Data S1 of the supplemental information and is publicly available as of the date of publication.
• Any additional information required to re-analyze the data reported in this study is available from the lead contact upon request.

## Acknowledgments

This research progressed over a 10-year period, with multiple visits to the Galápagos Islands that highlighted rapidly evolving biosecurity processes and the changing tourism pressures. During that time, we acknowledge the hospitality, vital discussions and thank the Charles Darwin Foundation, Galápagos Conservancy, Galápagos National Park Directorate (GNPD), the Galápagos Biosecurity Agency (ABG), the Ecuadorian Navy (DIRNEA), and the Navy’s Oceanographic Institute (INOCAR) for sharing their insights and biosecurity knowledge. We particularly thank I. Keith, C. Causton, and V. Toral (Charles Darwin Foundation), T. Dawson (University of Dundee), and K. Collins (University of Southampton) for many insightful discussions.

## Author contributions

M.L.C. conceptualized the research idea and collected and collated the data. All authors were involved in the formal analysis and data interpretation. M.L.C. and C.T.U.L. co-wrote the original draft. C.T.U.L. performed data presentation and visualization. All authors were involved in review and editing.

## Declaration of interests

The authors declare no competing interests.

## Declaration of generative AI and AI-assisted technologies in the writing process

Microsoft Co-Pilot (AI) was used to correct grammatical and syntax errors. The draft manuscript was written and edited by the authors. Co-pilot was used at the final stage to ensure grammatical and syntax errors were resolved prior to submission.

## STAR★Methods

### Key resources table

**Table:** 

REAGENT or RESOURCE	SOURCE	IDENTIFIER
**Software and algorithms**		

R Statistical Software v4.4.2	R Core Team,^72^https://www.R-project.org/	R v4.4.2
RStudio v2024.9.1.394	Posit Team,^73^http://www.posit.co/	RStudio v2024.9.1.394
R package ‘igraph’	Csardi and Nepusz,^74^https://igraph.org/	‘igraph’ R package version 2.1.4
R package ‘DirectedClustering’	Clemente,^75^https://CRAN.R-project.org/package=DirectedClustering	‘DirectedClustering’ R package version 1.0.0
R package ‘ggraph’	Pederson,^76^https://CRAN.R-project.org/package=ggraph	‘ggraph’ R package version 2.2.1
R package ‘leidenAlg’	Kharchenko et al.,^77^https://CRAN.R-project.org/package=leidenAlg	‘leidenAlg’ R package version 1.1.5

### Method details

#### Study site

##### Galápagos Islands at threat

The Galápagos Islands represent a unique opportunity to study a small-world network model for marine biosecurity and its relationship to tourism. The islands are recognized as a UNESCO World Heritage site and are facing various threats, including biosecurity, tourism, evolving governance arrangements, and solid waste.^78^ The small-world network model being studied is focused on the interlinked threat of biosecurity and tourism, as the growing tourism industry in the Galápagos Islands has a significant impact on the environment.

The history of the Galápagos Islands dates back to 1535 when it was discovered, but it remained uninhabited until 1833,^66^^,^^79^ when government incentives encouraged settlers to establish local fishing and industries.^54^ The introduction of air travel to the islands in 1968^80^ led to the development of the tourism industry, which has since become an important income source for the local population, stimulating economic and population growth.

Being inscribed as a World Heritage Site in 1978, the Galápagos Islands have increasingly become a destination for international tourism. Tourism in the Galápagos Islands started to take off in the 1970s, with the growth of liveaboard cruise vessels,^49^^,^^54^ but it has since shifted towards land-based tourism and island hopping.^81^^,^^82^ The number of tourists visiting the islands has expanded exponentially since the late 1970s.^49^ Early tourist growth was unregulated, leading to site competition and inter-island tourist operator conflicts.^7^^,^^54^^,^^83^ The growing tourism industry has provided an alternative livelihood for fishers and their families, especially when much of the local fisheries have been overexploited,^66^^,^^84^ made worse by the collapse of mainland Ecuadorian fisheries and the subsequent migration of fishers and others to the Galápagos.^83^ This growth has also led to conflicts and social issues between different stakeholders, including tourists, fishers, scientists, and park governance.

A socio-economic conundrum exists where communities are reliant upon healthy environmental ecosystems to attract tourism, but tourism can degrade the environment.^67^^,^^68^ The Galápagos Marine Reserve (GMR) was created in 1986 and extended in 1998 (https://whc.unesco.org/en/list/1) with the creation of the Galápagos Special Law.^85^ These legal instruments aim to protect biodiversity, endemism, and the geology of the region. Encompassed within the GMR aims, is a broad conceptualisation to improve management of marine resources, reduce resource conflicts, improve governance, and grow tourism opportunities,^7^^,^^81^^,^^86^ while balancing social-ecological needs and desires.^67^^,^^68^^,^^80^ More recent tourist growth has been regulated and aligned with spatial planning, sustainability, and ecotourism principles. Despite these efforts, the shift from fisheries to land-based tourism has yet to fully reconcile the social issues and consequently, hegemonic tension between fishers, tourism, scientists, and park governance remain an ongoing challenge.^67^^,^^69^^,^^86^^,^^87^

In 2007, the Galápagos World Heritage estate was listed as a heritage estate at risk, due to increasing introduced species, population growth, and tourism.^6^ By 2012, the Galápagos Marine Invasive Species project started to influence the understanding of NIMS presence and risks, leading to plans for international shipping into the GMR to be greatly reduced and re-routed through mainland Ecuador to Santa Cruz and now to San Cristobal. Of note, both marine and terrestrial ecosystems are affected by non-indigenous (alien) invasive species which created an external perception that the biosecurity system is inadequate (https://whc.unesco.org/en/soc/3450; https://whc.unesco.org/en/soc/4163; https://www.thegef.org/projects-operations/projects/9282;^65^^,^^66^).

Overall, the Galápagos Islands provide a unique and complex system for studying the relationship between marine biosecurity and tourism, and the challenges and opportunities for balancing social-ecological needs and desires in a semi-controlled system.

##### Galápagos Islands maritime connectivity

A rapid assessment of the international vessel connectivity of the Galápagos Islands conducted by the authors identifies connections with eight IUCN bioregions (Figure 1). Strong connections are observed with the southeast Pacific Ocean (including Chile, Ecuador, Peru, and Panama), the northeast Pacific Ocean (USA, Canada, extending to Mexico and Nicaragua), and the Wider Caribbean (including Costa Rica) based upon the last port of call and annual entry data.^88^^,^^89^ These connections are driven by cargo vessels, tourist cruise ships, fishing boats (legal and illegal), research vessels, naval patrol boats, and international yachts, representing primary pathways for the initial introduction of NIMS into the archipelago.^40^^,^^70^^,^^88^ NIMS can spread further through secondary introduction facilitated by inter-island vessel movements.

Within the archipelago (Figure 4), in the past and when this research occurred, Puerto Ayora on Santa Cruz Island served as the main maritime “hub” (central marine port transport location), with direct connection to mainland Ecuador for both incoming and outgoing shipping. Puerto Ayora is also the centre of commerce and economy for the archipelago.^49^ From this hub, domestic vessels transport tourists and goods to the different islands and attractions.^40^ Now, all vessels entering the region must transit via San Cristobal where they are inspected by the Galapagos Biosecurity Agency (https://bioseguridadgalapagos.gob.ec/). Inspections are focused on pests associated with terrestrial systems, and a dedicated marine team inspecting hull biofouling, but no evidence of sea-chests, intake/discharge pipelines (e.g., intake and discharge valves), or ballast pumps being inspected for marine invasive species (https://www.gob.ec/abg). This indicates a risk of unnoticed NIMS spreading across the connected islands once an island is infected.

#### Data collection

Our study focused on the domestic tourist small vessel traffic within the Galapagos archipelago. We used a one-year period of all scheduled (where a timetabled route was advertised online) domestic tourist small-vessel traffic (February 2015 to January 2016) to examine the connectivity between islands. Cargo vessels, recreational vessels, and liveaboard tourist vessels were excluded. The annual data was collected from tourist websites and catalogued to determine the departure and destination islands, frequency of trips, and number of connections to each island over this 12-month period.

To further enrich our model, sea surface temperature (SST) was selected as an environmental filter as it is known to limit species distributions.^90^^,^^91^^,^^92^^,^^93^ We obtained mean SST (the first day of each month between February 2015 and January 2016) for the primary tourist locations and ports on the 23 islands from the State of the Oceans dataset (SOTO; https://www.earthdata.nasa.gov/data/tools/soto). SOTO provides Group for High Resolution Sea Surface temperature (GHRSST) daily, at 1km Multiscale Ultrahigh Resolution (MUR) Level 4 sea surface temperature based upon night-time observations from multiple instruments (JPL MUR MEaSUREs Project. 2015.GHRSST Level 4 MUR Global Foundation Sea Surface Temperature Analysis (v4.1). Ver. 4.1. PO.DAAC, CA, USA, https://doi.org/10.5067/GHGMR-4FJ04). Temperature differences were then calculated for all pairs of islands each month and then categorised into 1°C temperature change categories.

### Quantification and statistical analysis

#### Domestic tourist small-vessel traffic as a directed weighted network

The domestic tourist small-vessel traffic represents a social network, both at the macro level (across the entire domestic tourist small-vessel network that we examined) and the micro level (between individual vessels and the locations they visit). In this analysis, each island, consisting of its associated ports, or tourist destinations that are visited by vessels, was treated as a node (i.e., vertex) in the network, with shipping routes acting as directed connections (i.e., edges) that transfer species from one island to another. We defined the edge weight as the frequency of trips between any pair of islands (nodes). The network was then viewed as a weighted directed network.

To explore the anthropogenic connectivity between each of the Galápagos islands, we used the ‘igraph’^74^ and ‘DirectedClustering’^75^ packages in the R environment (Version 4.4.2^72^). We first tested for the presence of a small-world network in the system, characterised by a small average path length and high clustering coefficient i.e., high likelihood a neighbour port becomes a feeder port.^50^^,^^94^^,^^95^^,^^96^ To do that, we calculated the small-world-ness index S, adapted from Humphries and Gurney (2008)^97^ to account for edge weights:

S = (C_g_/C_rand_)/(L_g_/L_rand_)

where:

C_g_: Global clustering coefficient of the observed network;

C_rand_: Global clustering coefficient of a random network;

L_g_: Average path length of the observed network;

L_rand_: Average path length of a random network.

In this calculation, the direction of the edges was disregarded, and the weight was the total number of trips between any pair of nodes. Metrics for the random network were averaged over 1,000 simulations, each of which matched the observed Galápagos Islands network in terms of node and edge counts. Edge weights were randomly sampled from the observed network’s weight distribution. As the edge weights represent the connection strength between nodes, they were inverted to depict the distances in the calculation of the weighted average/shortest path length (following Opsahl et al., 2010^98^). A network with S > 1 is deemed to be ‘small-world’.^97^

We examined the directed weighted network’s topology, specifically its average shortest (geodesic) path length, average clustering (i.e., tightness of the network), and modularity. These metrics have been used in previous studies on national airports,^99^ subway train systems,^100^^,^^101^ movement of maritime shipping,^94^^,^^102^^,^^103^ and water distribution systems.^104^ In the marine biosecurity management context, we were particularly interested in node-level statistics. Specifically, we computed each node’s out-degree (i.e., number of connections to other nodes, or its direct invasion diffusion range), out-strength (i.e., total weighted connections to other nodes, representing the intensity of its outbound connections to its neighbouring nodes), betweenness (i.e., bridge importance, or how often it sits on the shortest routes between other nodes), and out-closeness (i.e., how close it is to all other nodes along the shortest routes, implying how easily the invasion can transmit to other islands).^105^ The nodes/islands were then ranked based on these metrics.

We identified the presence of separate communities in the network (i.e., clusters of nodes where connections between the cluster’s members are stronger and denser than the connections between its members and other nodes). Communities imply that when an introduced species infects any member node of a community, it is more likely to spread within that community than to nodes outside of it. We used the Leiden algorithm^106^ for community detection in the weighted directed network via the ‘leidenAlg’ R package.^77^ For each identified community, we calculated the community’s ‘vulnerability metrics’, including community size (number of nodes), number of bridges (edges that connect to other communities), internal flow (i.e., total traffic within the community), total inflow (i.e., total incoming traffic from other communities), total outflow (total outgoing traffic from it to other community), in-closeness (averaged in-closeness of the community’s members, representing how easily infections in other communities can reach the community), out-closeness (averaged out-closeness of the community’s members, representing how easily infections within the community can spread to the others), and average vertex betweenness. The higher the metrics, the more vulnerable the community is to being infected by or spreading NIMS once a node is infected.

#### Domestic tourist small-vessel traffic – A network with SST-integrated composite weights

Higher similarity in SST between a pair of connected nodes indicates higher habitat suitability and greater survival of introduced species. In this analysis, we calculated SST similarity (S_SST_) as a Gaussian kernel of the absolute SST difference as follows:

$$\text{SSST}={\mathrm{e}}^{-\left(\frac{\Delta T^{2}}{2\sigma_{T}^{2}}\right)}$$

where Δ*T*is the absolute SST difference between a pair of nodes, and σ_T_ = 2, is the temperature scale in degrees Celsius to control how fast the similarity drops when Δ*T* increases (as suggested by Seebens et al., 2013^107^). Accordingly, S_SST ∈_ (0, 1] and decreases smoothly when Δ*T* increases. We calculated S_SST_ for each month between February 2015 and January 2016. To identify the month with the highest mean S_SST_, we compared S_SST_ across the 12 months using a Kruskal-Wallis test for non-normal data (as indicated by our preliminary analysis), followed by post-hoc pairwise Dunn’s tests with the Benjamini-Hochberg p-value adjustment to control for false discovery rate^108^ in multiple testing. Statistical significance levels of the results are indicated by asterisks as follows: ∗*p* < 0.05, ∗∗*p* < 0.01, ∗∗∗*p* < 0.001.

We integrated SST into the network by computing a composite weight matrix, which is the product of the frequency of trips and SST similarity between a pair of nodes. The composite weight tells us that any pair of nodes (islands) with a greater frequency of trips and higher similarity in SST has a stronger connection or higher proximity in the introduced species context. The concept of composite weight has been applied in various areas, such as ecological connectivity,^109^^,^^110^ predictive marine bioinvasions caused by global shipping,^107^ and epidemiologic contact networks.^111^ The same network statistics as previously introduced were calculated using the composite weight for each month. For illustrative purposes, we present the result for only the month that had the highest mean SST similarity.

The network was visualised with nodes placed relative to the islands’ geographic locations, with some adjustments made for visibility where the nodes were too clustered. The ‘ggraph’ R package^76^ was used for this visualisation.

## Supplemental information

Document S1. Figures S1−S3, Tables S1−S5, and Data S1
