# Supplementary material for: Protecting Galápagos’ marine ecosystems: Biosecurity and network design against invasive species from tourist vessels
Source: iScience. 2026 Mar 26;29(5):115486. doi: 10.1016/j.isci.2026.115486 (PMC13138223; doi:10.1016/j.isci.2026.115486)
Supplement: Document S1. Figures S1−S3, Tables S1−S5, and Data S1 [file mmc1.pdf]

iScience, Volume *29*

Supplemental information

Protecting Galápagos' marine ecosystems: Biosecurity and network design against invasive species from tourist vessels

Marnie L. Campbell, Chi T.U. Le, and Chad L. Hewitt

SUPPLEMENTAL MATERIALS

This supplementary information has not been peer reviewed.

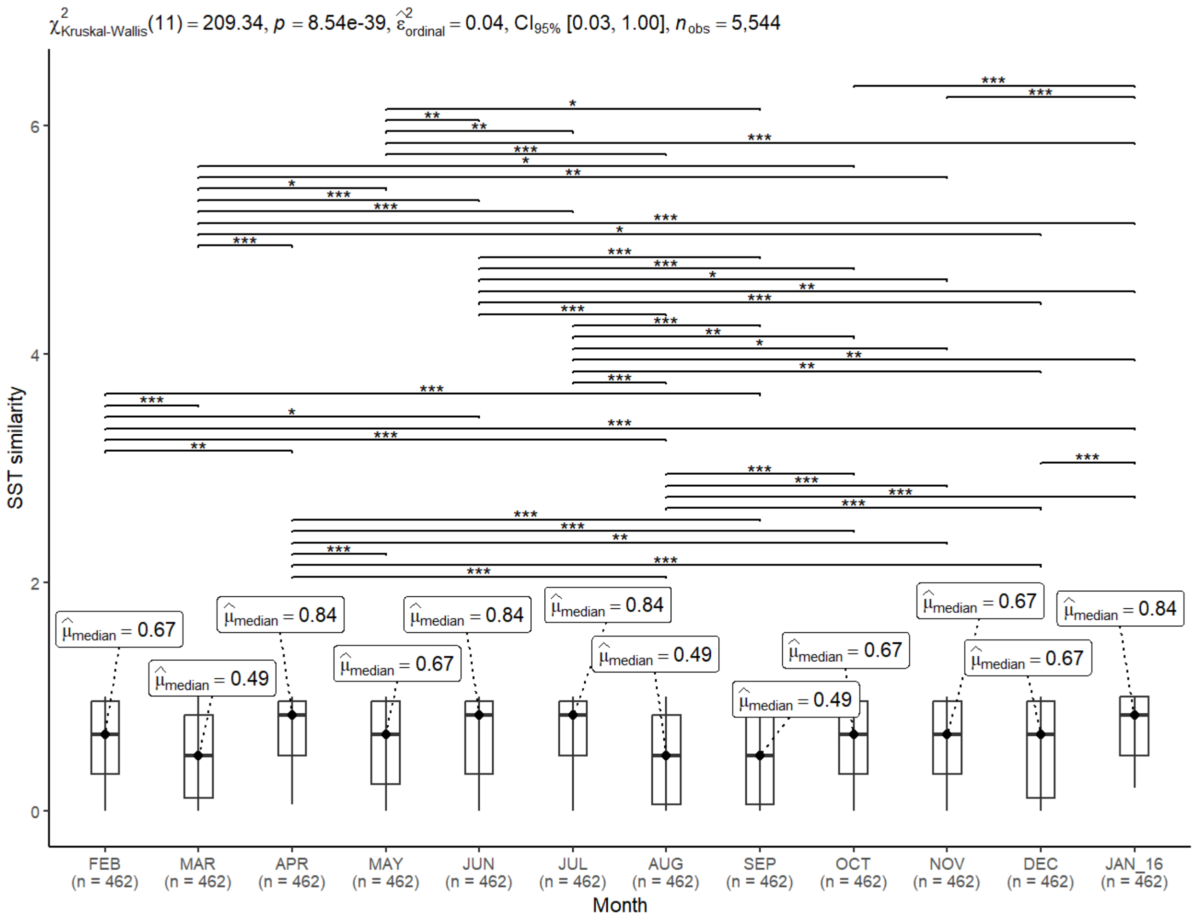

Figure S1: Pairwise comparison of sea surface temperature (SST) similarity across months during the study period (February 2015 to January 2016). Asterisks denote significance thresholds for Benjamini–Hochberg–adjusted p-values from post hoc pairwise comparisons using Dunn's test: * $p < 0.05$, ** $p < 0.01$, *** $p < 0.001$.

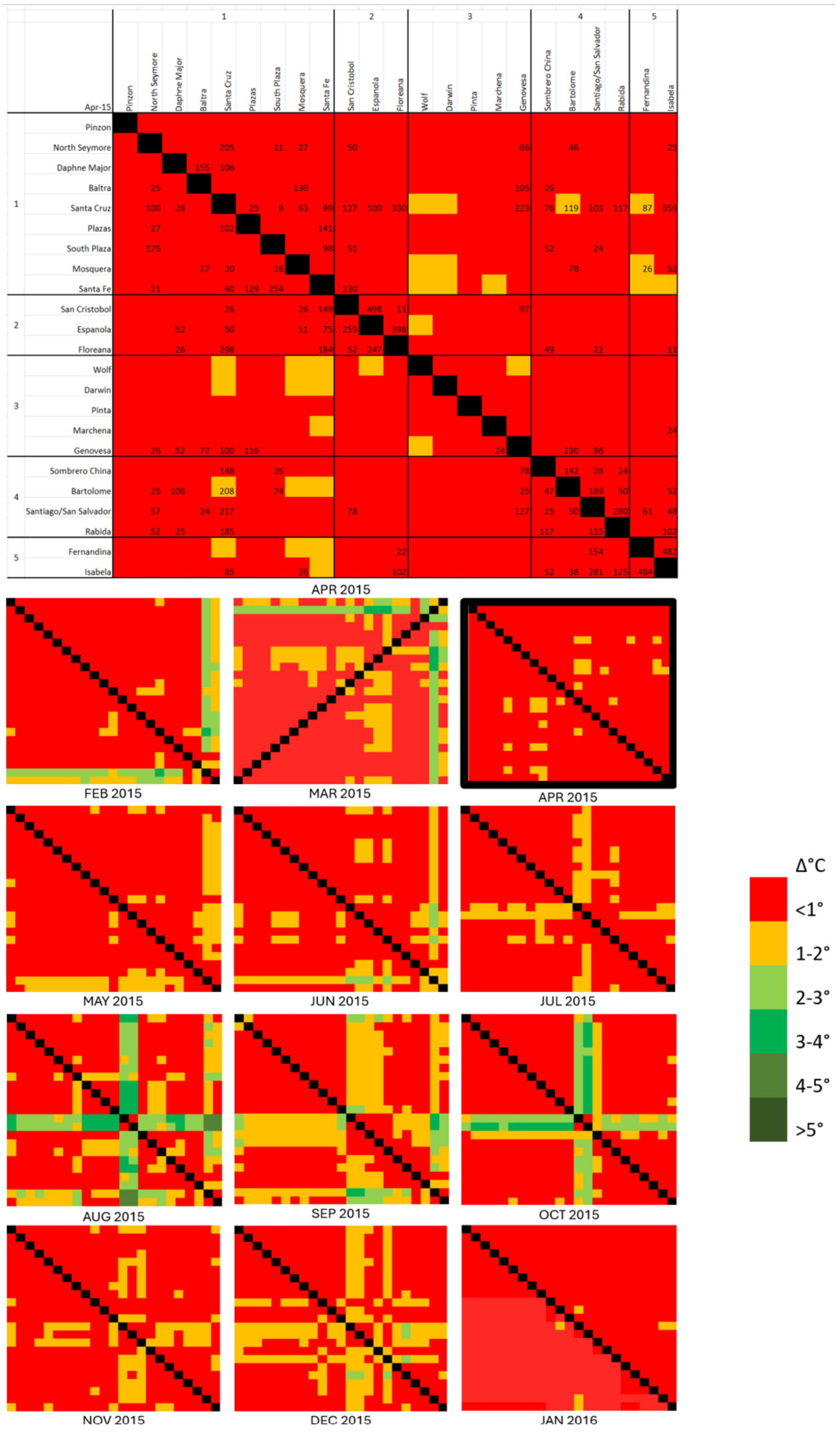

Figure S2: Mean island sea surface temperature (SST) differences for all pairwise island combinations (numbers on the outside represent the Harris SST zones; Harris 1969) by month (April 2015 expanded to illustrate detail) with annual number of tourist vessel connections. Colours represent absolute temperature differences between locations in 1°C difference categories.

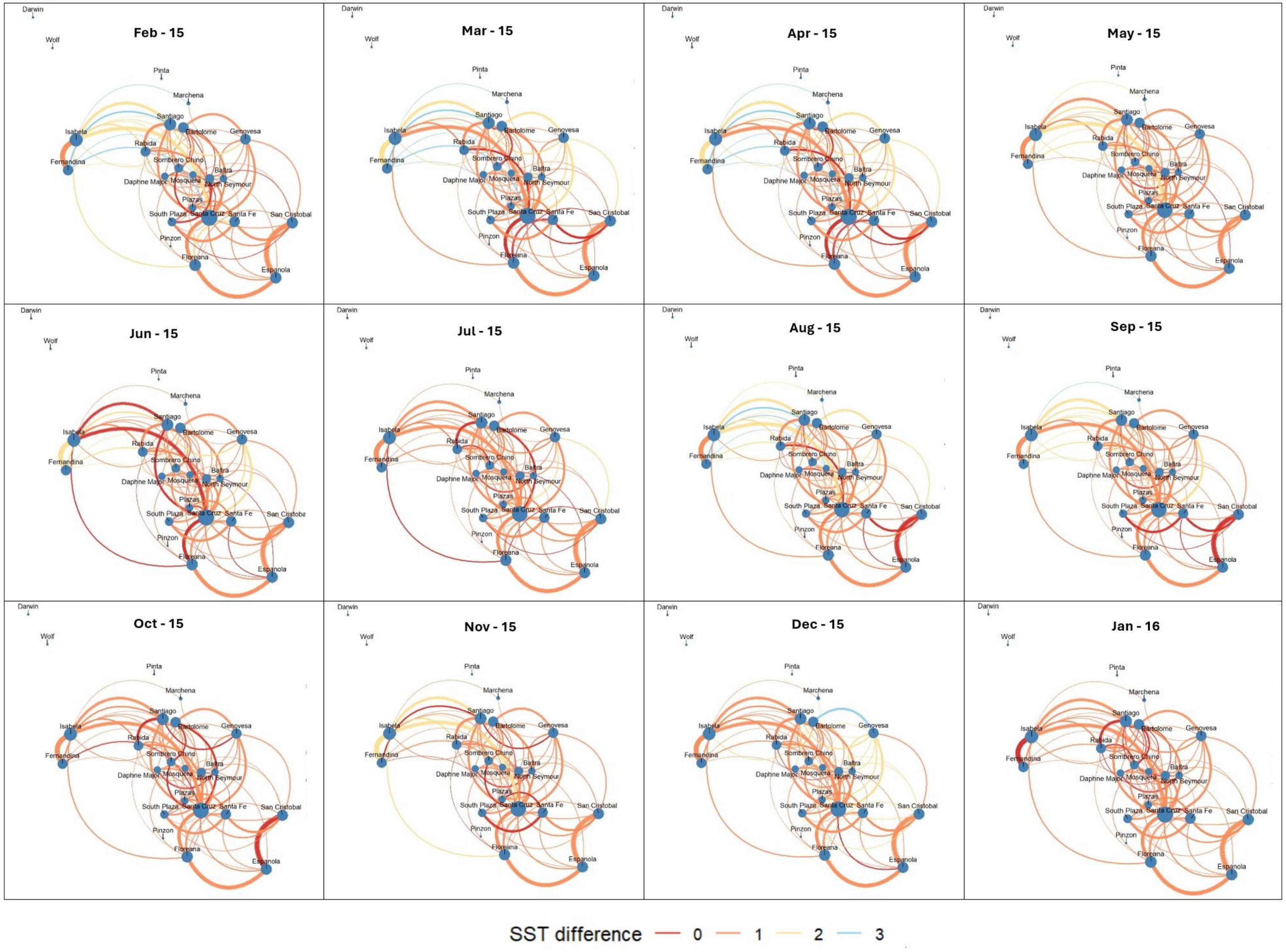

Figure S3: SST difference among islands in the Galapagos archipelago across the months from February 2015 to January 2016.

Table S1: Examples of tourist activities accessed by tourism small-vessels at each of the Galápagos Islands grouped into the five Harris (1969) sea surface temperature zones. Grey denotes that the activity is present at that island and in that Harris zone. White denotes a lack of presence of that activity (at the time that these analyses occurred).

Harris zone	Activity	Diving	Snorkelling	Sea turtle	Shark	Hammerhead shark	Colourful fish	Flightless cormorant	Frigate	Blue footed boobie	Red footed boobie	Masked boobie	Flamingo	Albatross	Penguin	Heron	Swallow tailed gulls	Various birds	Giant tortoise	Land iguana	Marine iguana	Dolphins	Whales	Sea lion	Fur seal
	**Pinzon Island**		■	■		■									■				■		■	■		■	
	**North Seymour Island**	■							■	■											■			■	
	**Daphne Major**	■															■								
	**Baltra**	■	■														■								
1	**Santa Cruz Island**	■																	■		■			■	
	**Plazas Island**									■							■			■	■			■	
	**South Plaza Island**																			■	■			■	
	**Mosquera Island**		■	■	■		■											■						■	
	**Santa Fe Island**	■	■																	■	■			■	
	**San Cristobal Island**	■	■						■	■	■								■					■	
2	**Espanola Island**	■			■		■			■				■						■	■			■	
	**Floreana Island**	■	■	■			■			■		■	■		■									■	
	**Wolf**	■																					■		
	**Darwin Island**	■																					■		
3	**Pinta Island**																■				■				■
	**Marchena Island**	■			■	■	■			■												■		■	■
	**Genovesa Island**	■	■		■	■			■	■	■	■					■	■			■				
	**Sombrero Chino Island**														■									■	
4	**Bartolome Island**			■	■										■									■	
	**Santiago Island**	■		■			■						■			■				■	■			■	■
	**Rabida Island**	■											■											■	
5	**Fernandina Island**		■					■							■						■	■	■	■	
	**Isabela Island**	■	■	■	■			■					■		■				■	■	■	■	■	■	

Table S2: Proportion of possible vessel connections that are currently used by tourist vessels within and between Harris (1969) zones 1 – 5 as illustrated by the zones in Figure 1. Please note that the number in parentheses denotes the number of islands identified within the zone.

**Origin**	**Destination** 1 (9)	2 (3)	3 (5)	4 (4)	5 (2)
1 (9)	0.36	0.22	0.07	0.25	0.28
2 (3)	0.37	1.00	0.07	0.17	0.17
3 (5)	0.11	0.00	0.10	0.10	0.10
4 (4)	0.33	0.08	0.15	0.86	0.50
5 (2)	0.11	0.33	0.00	0.63	1.00

Table S3: Network statistics for each node in the Galapagos Islands weighted network in descending order of node strength (in).

	Island	In-degree	Out-degree	Strength (in)	Strength (out)	Between-ness	Closeness (in)	Closeness (out)
1	Santa Cruz	**14**	**16**	**1810**	**1963**	**0.56**	**90.31**	**86.60**
2	Isabela	9	8	1155	1193	0.19	82.49	59.42
3	Santiago	9	10	990	967	0.13	71.45	68.50
4	Floreana	5	8	863	889	0.20	79.24	75.16
5	San Cristobal	7	6	847	807	0.05	61.21	63.63
6	Espanola	3	6	845	845	0.06	64.32	66.76
7	Santa Fe	6	5	746	694	0.08	62.28	56.42
8	Genovesa	7	8	721	721	0.22	72.50	63.88
9	Bartolome	7	9	703	756	0.17	61.73	70.98
10	Fernandina	4	3	658	658	0.00	71.06	53.22
11	Rabida	5	6	596	596	0.01	61.23	63.48
12	North Seymour	9	7	508	430	0.04	52.57	63.01
13	Sombrero Chino	8	6	444	444	0.00	46.15	60.27
14	South Plaza	6	5	400	400	0.01	53.14	51.86
15	Daphne Major	6	2	287	261	0.01	41.53	54.84
16	Baltra	4	4	283	286	0.01	41.10	43.09
17	Mosquera	6	6	283	229	0.00	42.35	39.02
18	Plazas	3	3	270	270	0.01	50.44	53.55
19	Marchena	1	1	24	24	0.00	19.04	18.00
20	Darwin	0	0	0	0	-	-	-
21	Pinta	0	0	0	0	-	-	-
22	Pinzon	0	0	0	0	-	-	-
23	Wolf	0	0	0	0	-	-	-
	Average	*6.26*	*6.26*	*654.37*	*654.37*	*0.09*	*59.17*	*58.51*

Note: CC – clustering coefficient. Bold numbers represent the highest values in individual metrics. (-): not available.

Table S4: Comparison matrix of sea surface temperature (SST) similarity among the 12 months between February 2015 and January 2016. Numbers in parentheses below month labels indicate monthly SST similarity medians. Cell values represent Dunn's test Z statistics, with significant results indicated by grey shading and bold text (ns: non-significant, * $p < 0.05$, ** $p < 0.01$, *** $p < 0.001$).

Month	FEB (0.67)	MAR (0.49)	APR (0.84)	MAY (0.67)	JUN (0.84)	JUL (0.84)	AUG (0.49)	SEP (0.67)	OCT (0.67)	NOV (0.67)	DEC (0.67)	JAN 16 (0.84)
**FEB (0.67)**		1.28 (ns)	0.23 (ns)	0.20 (ns)	**3.30 (**)**	2.00 (ns)	1.46 (ns)	0.54 (ns)	1.68 (ns)	1.24 (ns)	1.50 (ns)	**2.72 (*)**
**MAR (0.49)**			1.05 (ns)	1.08 (ns)	**4.58 (***)**	**3.28 (***)**	0.18 (ns)	1.82 (ns)	**2.96 (*)**	**2.52 (*)**	0.22 (ns)	**4.00 (***)**
**APR (0.84)**				0.03 (ns)	**3.53 (**)**	2.23 (ns)	1.23 (ns)	0.77 (ns)	1.91 (ns)	1.47 (ns)	1.27 (ns)	**2.95 (*)**
**MAY (0.67)**					**3.49 (*)**	2.19 (ns)	1.27 (ns)	0.74 (ns)	1.88 (ns)	1.44 (ns)	1.30 (ns)	**2.91 (*)**
**JUN (0.84)**						1.30 (ns)	**4.76 (***)**	**2.76 (*)**	1.61 (ns)	2.05 (ns)	**3.46 (***)**	**4.79 (***)**
**JUL (0.84)**							**3.46 (**)**	1.46 (ns)	0.32 (ns)	0.75 (ns)	**3.50 (***)**	0.72 (ns)
**AUG (0.49)**								2.00 (ns)	**3.14 (**)**	**2.70 (*)**	0.04 (ns)	**4.18 (***)**
**SEP (0.67)**									1.14 (ns)	0.70 (ns)	2.04 (ns)	2.18 (ns)
**OCT (0.67)**										0.44 (ns)	**3.18 (**)**	1.04 (ns)

Table S5: Network statistics for each node in the Galapagos Islands directed weighted network in descending order of node strength (in) for April with SST similarity incorporated

	Island	Strength (in)	Strength (out)	Betweennes s	Closenes s (in)	Closeness (out)
1	Santa Cruz	**925.21**	**1075.45**	**0.43**	**49.63**	**42.63**
2	Espanola	736.43	659.33	0.04	39.57	33.99
3	Isabela	730.59	904.17	0.16	48.28	36.61
4	San Cristobal	701.04	720.82	0.06	38.84	35.06
5	Santiago	669.16	771.60	0.17	43.02	39.13
6	Genovesa	574.74	408.75	0.25	17.10	35.79
7	Floreana	535.14	514.89	0.06	46.09	35.75
8	Fernandina	504.85	550.42	0.00	18.03	34.06
9	Rabida	458.49	443.51	0.03	38.69	38.38
10	Santa Fe	394.00	339.26	0.07	38.09	31.56
11	North Seymour	366.44	213.63	0.04	33.70	28.45
12	Sombrero Chino	330.83	267.20	0.03	32.16	29.11
13	Bartolome	284.93	374.12	0.07	31.79	33.55
14	Baltra	235.79	217.01	0.03	23.91	27.01
15	Mosquera	195.85	72.42	0.00	29.30	13.94
16	Daphne Major	189.13	163.88	0.01	27.02	23.79
17	South Plaza	184.23	314.37	0.03	33.01	27.32
18	Plazas	131.42	121.24	0.00	28.52	24.39
19	Marchena	7.79	24.00	0.00	10.15	16.39
20	Darwin	0	0	-	-	-
21	Pinta	0	0	-	-	-
22	Pinzon	0	0	-	-	-
23	Wolf	0	0	-	-	-
	Average	*429.27*	*429.27*	*0.08*	*32.99*	*30.89*

Note: CC – clustering coefficient. Bold numbers represent the highest values in individual metrics. (-): not available

Data S1: R Code for network analysis

```
library(sf)
library(ggplot2)
library(tidygraph)
library(dplyr)
library(igraph)
library(ggraph)
library(ggstatsplot)
library(DirectedClustering)
library(RColorBrewer)
library(statsExpressions)
library(leidenAlg)

#==============================================================#
#              Data preparation                    #
#==============================================================#

# Galapagos data:

island_data <- read.csv("SST_data.csv", header=TRUE)

island_coords <- island_data %>% select(name, harris_region, lat, long)

voyages <- read.csv("Edges.csv", header=TRUE)
# Remove " Island" in the dataframe of voyages to match with island data:

voyages[] <- lapply(voyages, function(x){
  if (is.character(x)){
    sub(" Island$", "", x)
  }
```

```
  else {x}
})


# Convert to spatial points (if needed to overlay on a map):

# island_sf <- st_as_sf(island_coords, coords = c("long", "lat"), crs=st_crs(4326))

# Manually adjust positions to avoid overlapping islands near Santa Cruz:
islands_adj <- island_coords %>%
  mutate(
    lat_adj = case_when(
      name == "Santa Cruz" ~ lat,
      name == "Baltra" ~ lat + 0.1,
      name == "Mosquera" ~ lat + 0.1,
      name == "Daphne Major" ~ lat + 0.1,
      name == "North Seymore" ~ lat + 0.2,
      name == "Plazas" ~ lat - 0.1,
      name == "South Plaza" ~ lat - 0.2,
      name == "Rabida" ~ lat + 0.3,
      name == "Pinzon" ~ lat - 0.4,
      name == "Sombrero Chino" ~ lat + 0.1,
      name == "Santiago" ~ lat + 0.5,
      name == "Bartolome" ~ lat + 0.2,
      name == "Darwin" ~ lat - 0.5,
      name == "Wolf" ~ lat - 0.5,
      TRUE ~ lat
    ),
    lon_adj = case_when(
      name == "Santa Cruz" ~ long,
      name == "Baltra" ~ long - 0.1,
```

```
    name == "Mosquera" ~ long + 0.2,
    name == "Daphne Major" ~ long + 0.4,
    name == "North Seymore" ~ long - 0.4,
    name == "Plazas" ~ long + 0.3,
    name == "South Plaza" ~ long + 0.5,
    name == "Rabida" ~ long + 0.2,
    TRUE ~ long
   )
  )


edges <- voyages %>%
  left_join(islands_adj, by = c("from" = "name")) %>%
  dplyr::rename(x_from = lon_adj, y_from = lat_adj) %>%
  left_join(islands_adj, by = c("to" = "name")) %>%
  dplyr::rename(x_to = lon_adj, y_to = lat_adj)

# Remove edges with identical start and end points:
edges <- edges %>%
  filter(from != to)



##======== Incorporating SST differences =========##

island_sst <- island_data %>% select(-bioregion, -harris_region, -lat, -long)
sst_diff <- edges %>%
  left_join(island_sst, by = c("from"="name")) %>%
  rename_with(~ paste0(.x, "_from"), matches("^[a-z]{3}(_\\d+)?$")) %>%
  left_join(island_sst, by = c("to" = "name")) %>%
  rename_with(~ paste0(.x, "_to"), matches("^[a-z]{3}(_\\d+)?$"))
```

```
months <- c("feb", "mar", "apr", "may", "jun", "jul", "aug", "sep", "oct", "nov", "dec", "jan_16")
length_scale <- 2 # sigma T for calculating temperature similarity

for (month in months) {
 from_col <- paste0(month, "_from")
 to_col <- paste0(month, "_to")
 diff_col <- paste0("diff_", month)
 diff_category <- paste0("diff_cat_", month)
 sim_col <- paste0("sim_", month)
  sst_diff[[diff_col]] <- abs(sst_diff[[from_col]] - sst_diff[[to_col]])
 sst_diff[[diff_category]] <- cut(sst_diff[[diff_col]],
                 breaks = c(-Inf, 0, 1, 2, 3, 4, 5, Inf),
                 labels = 0:6,
                 right = TRUE)
 sst_diff[[sim_col]] <- exp(-(sst_diff[[diff_col]]^2/2*length_scale^2))
}

temp_diff <- sst_diff %>% select(from, to, contains("diff"), contains("sim"))
summary(temp_diff)

# select(sst_diff, contains("diff")) %>% max(): maximum difference is 4.8 Celsius degrees

edges <- edges %>% left_join(temp_diff, by = c("from"="from", "to"="to"))

##======== Create a network object ============##

#------------- Undirected graph ----------------#

g <- graph_from_data_frame(d = edges, vertices = islands_adj, directed = FALSE)

# reciprocity(g, mode = "ratio") => 1: all mutual connection
```

```
g_undirected <- as_undirected(g, mode = "collapse")

# Node weighted degrees (strength) (weights = no. of connection are the same with all islands
instead of Wolf):

strengths <- strength(g_undirected, weights = E(g_undirected)$freq)
V(g_undirected)$strength <- strengths


g_cleaned <- delete_edges(g_undirected, E(g_undirected)[freq == 0])

V(g_cleaned)$name
# Plot using ggraph:

# Using islands_adjust is the layout, and edge color corresponding to connection frequency:
ggraph(g_cleaned, layout = "manual", x = islands_adj$lon_adj, y = islands_adj$lat_adj) +
  geom_edge_arc(aes(edge_width = freq, edge_colour = freq), strength = 0.7, alpha = 0.8) +
  scale_edge_width(range = c(0.5, 5) , name = "") +
  scale_edge_colour_gradient(low = "lightblue", high = "darkred", name = "Connection
frequency") +
  geom_node_point(aes(size = strength), color = "steelblue") +
  scale_size(range=c(3, 15), name = "Node strength", guide = guide_legend(order = 1)) +
  geom_node_text(aes(label = name), repel=TRUE, vjust = -1.2, size = 5) +
  scale_x_reverse() +
  coord_fixed() +
  theme_void() +
  theme(legend.position = "right")

# Feb 15, and edge color corresponding to sst difference:
ggraph(g_cleaned_directed, layout = "manual", x = islands_adj$lon_adj, y = islands_adj$lat_adj)
+
```

```
 geom_edge_arc(aes(edge_width = freq, edge_colour = diff_cat_feb), strength = 0.6, alpha = 0.8)
+
 scale_edge_width(range = c(0.5, 5) , name = "Connection frequency") +
 scale_edge_colour_manual(values = c("0" = "#d73027", "1" = "#fc8d59", "2" = "#fee090",
                     "3" = "skyblue", "4" = "blue", "5" = "darkblue", "6"="black"),
               name = "SST difference") +
 geom_node_point(aes(size = strength), color = "steelblue") +
 scale_size(range=c(2, 15), name = "Node strength", guide = guide_legend(order = 1)) +
 geom_node_text(aes(label = name), repel=TRUE, vjust = -1.2, size = 5) +
 scale_x_reverse() +
 coord_fixed() +
 theme_void() +
 theme(legend.position = "right")


# Mar 15, and edge color corresponding to sst difference:
ggraph(g_cleaned, layout = "manual", x = islands_adj$lon_adj, y = islands_adj$lat_adj) +
 geom_edge_arc(aes(edge_width = freq, edge_colour = diff_cat_mar), strength = 0.6, alpha =
0.8) +
 scale_edge_width(range = c(0.5, 5) , name = "Connection frequency") +
 scale_edge_colour_manual(values = c("0" = "#d73027", "1" = "#fc8d59", "2" = "#fee090",
                     "3" = "skyblue", "4" = "blue", "5" = "darkblue"),
               name = "SST difference") +
 geom_node_point(aes(size = strength), color = "steelblue") +
 scale_size(range=c(2, 15), name = "Node strength", guide = guide_legend(order = 1)) +
 geom_node_text(aes(label = name), repel=TRUE, vjust = -1.2, size = 5) +
 scale_x_reverse() +
 coord_fixed() +
 theme_void() +
 theme(legend.position = "right")

# Apr 15, and edge color corresponding to sst difference:
```

```
ggraph(g_cleaned, layout = "manual", x = islands_adj$lon_adj, y = islands_adj$lat_adj) +
  geom_edge_arc(aes(edge_width = freq, edge_colour = diff_cat_apr), strength = 0.6, alpha = 0.8)
+
  scale_edge_width(range = c(0.5, 5) , name = "Connection frequency") +
  scale_edge_colour_manual(values = c("0" = "#d73027", "1" = "#fc8d59", "2" = "#fee090",
                                      "3" = "skyblue", "4" = "blue", "5" = "darkblue"),
                           name = "SST difference") +
  geom_node_point(aes(size = strength), color = "steelblue") +
  scale_size(range=c(2, 15), name = "Node strength", guide = guide_legend(order = 1)) +
  geom_node_text(aes(label = name), repel=TRUE, vjust = -1.2, size = 5) +
  scale_x_reverse() +
  coord_fixed() +
  theme_void() +
  theme(legend.position = "right")



# May 15, and edge color corresponding to sst difference:
ggraph(g_cleaned, layout = "manual", x = islands_adj$lon_adj, y = islands_adj$lat_adj) +
  geom_edge_arc(aes(edge_width = freq, edge_colour = diff_cat_may), strength = 0.6, alpha =
0.8) +
  scale_edge_width(range = c(0.5, 5) , name = "Connection frequency") +
  scale_edge_colour_manual(values = c("0" = "#d73027", "1" = "#fc8d59", "2" = "#fee090",
                                      "3" = "skyblue", "4" = "blue", "5" = "darkblue"),
                           name = "SST difference") +
  geom_node_point(aes(size = strength), color = "steelblue") +
  scale_size(range=c(2, 15), name = "Node strength", guide = guide_legend(order = 1)) +
  geom_node_text(aes(label = name), repel=TRUE, vjust = -1.2, size = 5) +
  scale_x_reverse() +
  coord_fixed() +
  theme_void() +
  theme(legend.position = "right")
```

```
# Jun 15, and edge color corresponding to sst difference:
ggraph(g_cleaned, layout = "manual", x = islands_adj$lon_adj, y = islands_adj$lat_adj) +
  geom_edge_arc(aes(edge_width = freq, edge_colour = diff_cat_jun), strength = 0.6, alpha = 0.8)
+
  scale_edge_width(range = c(0.5, 5) , name = "Connection frequency") +
  scale_edge_colour_manual(values = c("0" = "#d73027", "1" = "#fc8d59", "2" = "#fee090",
                                      "3" = "skyblue", "4" = "blue", "5" = "darkblue"),
                          name = "SST difference") +
  geom_node_point(aes(size = strength), color = "steelblue") +
  scale_size(range=c(2, 15), name = "Node strength", guide = guide_legend(order = 1)) +
  geom_node_text(aes(label = name), repel=TRUE, vjust = -1.2, size = 5) +
  scale_x_reverse() +
  coord_fixed() +
  theme_void() +
  theme(legend.position = "right")

# July 15, and edge color corresponding to sst difference:
ggraph(g_cleaned, layout = "manual", x = islands_adj$lon_adj, y = islands_adj$lat_adj) +
  geom_edge_arc(aes(edge_width = freq, edge_colour = diff_cat_jul), strength = 0.6, alpha = 0.8)
+
  scale_edge_width(range = c(0.5, 5) , name = "Connection frequency") +
  scale_edge_colour_manual(values = c("0" = "#d73027", "1" = "#fc8d59", "2" = "#fee090",
                                      "3" = "skyblue", "4" = "blue", "5" = "darkblue"),
                          name = "SST difference") +
  geom_node_point(aes(size = strength), color = "steelblue") +
  scale_size(range=c(2, 15), name = "Node strength", guide = guide_legend(order = 1)) +
  geom_node_text(aes(label = name), repel=TRUE, vjust = -1.2, size = 5) +
  scale_x_reverse() +
  coord_fixed() +
  theme_void() +
  theme(legend.position = "right")
```

August 15, and edge color corresponding to sst difference:

```
ggraph(g_cleaned, layout = "manual", x = islands_adj$lon_adj, y = islands_adj$lat_adj) +
  geom_edge_arc(aes(edge_width = freq, edge_colour = diff_cat_aug), strength = 0.6, alpha =
0.8) +
  scale_edge_width(range = c(0.5, 5) , name = "Connection frequency") +
  scale_edge_colour_manual(values = c("0" = "#d73027", "1" = "#fc8d59", "2" = "#fee090",
                                      "3" = "skyblue", "4" = "blue", "5" = "darkblue","6"="black"),
                           name = "SST difference") +
  geom_node_point(aes(size = strength), color = "steelblue") +
  scale_size(range=c(2, 15), name = "Node strength", guide = guide_legend(order = 1)) +
  geom_node_text(aes(label = name), repel=TRUE, vjust = -1.2, size = 5) +
  scale_x_reverse() +
  coord_fixed() +
  theme_void() +
  theme(legend.position = "bottom")
```

Sept 15, and edge color corresponding to sst difference:

```
ggraph(g_cleaned, layout = "manual", x = islands_adj$lon_adj, y = islands_adj$lat_adj) +
  geom_edge_arc(aes(edge_width = freq, edge_colour = diff_cat_sep), strength = 0.6, alpha =
0.8) +
  scale_edge_width(range = c(0.5, 5) , name = "Connection frequency") +
  scale_edge_colour_manual(values = c("0" = "#d73027", "1" = "#fc8d59", "2" = "#fee090",
                                      "3" = "skyblue", "4" = "blue", "5" = "darkblue"),
                           name = "SST difference") +
  geom_node_point(aes(size = strength), color = "steelblue") +
  scale_size(range=c(2, 15), name = "Node strength", guide = guide_legend(order = 1)) +
  geom_node_text(aes(label = name), repel=TRUE, vjust = -1.2, size = 5) +
  scale_x_reverse() +
  coord_fixed() +
  theme_void() +
  theme(legend.position = "right")
```

```
# Oct 15, and edge color corresponding to sst difference:
ggraph(g_cleaned, layout = "manual", x = islands_adj$lon_adj, y = islands_adj$lat_adj) +
  geom_edge_arc(aes(edge_width = freq, edge_colour = diff_cat_oct), strength = 0.6, alpha = 0.8)
+
  scale_edge_width(range = c(0.5, 5) , name = "Connection frequency") +
  scale_edge_colour_manual(values = c("0" = "#d73027", "1" = "#fc8d59", "2" = "#fee090",
                          "3" = "skyblue", "4" = "blue", "5" = "darkblue"),
                    name = "SST difference") +
  geom_node_point(aes(size = strength), color = "steelblue") +
  scale_size(range=c(2, 15), name = "Node strength", guide = guide_legend(order = 1)) +
  geom_node_text(aes(label = name), repel=TRUE, vjust = -1.2, size = 5) +
  scale_x_reverse() +
  coord_fixed() +
  theme_void() +
  theme(legend.position = "right")

# Nov 15, and edge color corresponding to sst difference:
ggraph(g_cleaned, layout = "manual", x = islands_adj$lon_adj, y = islands_adj$lat_adj) +
  geom_edge_arc(aes(edge_width = freq, edge_colour = diff_cat_nov), strength = 0.6, alpha =
0.8) +
  scale_edge_width(range = c(0.5, 5) , name = "Connection frequency") +
  scale_edge_colour_manual(values = c("0" = "#d73027", "1" = "#fc8d59", "2" = "#fee090",
                          "3" = "skyblue", "4" = "blue", "5" = "darkblue"),
                    name = "SST difference") +
  geom_node_point(aes(size = strength), color = "steelblue") +
  scale_size(range=c(2, 15), name = "Node strength", guide = guide_legend(order = 1)) +
  geom_node_text(aes(label = name), repel=TRUE, vjust = -1.2, size = 5) +
  scale_x_reverse() +
  coord_fixed() +
  theme_void() +
  theme(legend.position = "right")
```

```
# Dec 15, and edge color corresponding to sst difference:
ggraph(g_cleaned, layout = "manual", x = islands_adj$lon_adj, y = islands_adj$lat_adj) +
  geom_edge_arc(aes(edge_width = freq, edge_colour = diff_cat_dec), strength = 0.6, alpha = 0.8) +
  scale_edge_width(range = c(0.5, 5) , name = "Connection frequency") +
  scale_edge_colour_manual(values = c("0" = "#d73027", "1" = "#fc8d59", "2" = "#fee090",
                                      "3" = "skyblue", "4" = "blue", "5" = "darkblue"),
                           name = "SST difference") +
  geom_node_point(aes(size = strength), color = "steelblue") +
  scale_size(range=c(2, 15), name = "Node strength", guide = guide_legend(order = 1)) +
  geom_node_text(aes(label = name), repel=TRUE, vjust = -1.2, size = 5) +
  scale_x_reverse() +
  coord_fixed() +
  theme_void() +
  theme(legend.position = "right")


# Jan 16, and edge color corresponding to sst difference:
ggraph(g_cleaned, layout = "manual", x = islands_adj$lon_adj, y = islands_adj$lat_adj) +
  geom_edge_arc(aes(edge_width = freq, edge_colour = diff_cat_jan_16), strength = 0.6, alpha = 0.8) +
  scale_edge_width(range = c(0.5, 5) , name = "Connection frequency", guide = guide_legend(order = 2)) +
  scale_edge_colour_manual(values = c("0" = "#d73027", "1" = "#fc8d59", "2" = "#fee090",
                                      "3" = "skyblue", "4" = "blue", "5" = "darkblue"),
                           name = "SST difference", guide = guide_legend(order = 3)) +
  geom_node_point(aes(size = strength), color = "steelblue") +
  scale_size(range=c(2, 15), name = "Node strength", guide = guide_legend(order = 1)) +
  geom_node_text(aes(label = name), repel=TRUE, vjust = -1.2, size = 5) +
  scale_x_reverse() +
  coord_fixed() +
  theme_void() +
  theme(legend.position = "right")
```

```
##====== Examining whether Galapagos network is small-world =====#

# Simplifying the graph by removing multiple edges and combining weights:
g_simple <- simplify(g_cleaned, remove.multiple = TRUE, remove.loops = TRUE,
            edge.attr.comb = list(freq="sum"))

weights <- E(g_simple)$freq
V(g_simple)$name

# C_obs <- mean(transitivity(g_simple, type = "weighted", weights = weights), na.rm = TRUE)
# same result with the below function

# Observed global weighted clustering coefficient (ClustBCG function of DirectedClustering R package)

# Adjacency matrix from g_simple:

A <- as_adjacency_matrix(g_simple, sparse = FALSE, attr = "freq")
C_obs <- ClustBCG(A, "undirected", isolates = "NaN")$GlobalCC

# Observed  weighted average path length: # Weights representing frequency, so inver
L_obs <- mean_distance(g_simple, weights = 1/weights)

# simulate random weighted networks with the same numbers of nodes and edges
n_nodes <- gorder(g_simple)
n_edges <- gsize(g_simple)

n_sim <- 1000
C_rand <- numeric(n_sim)
L_rand <- numeric(n_sim)
```

```
for (i in 1:n_sim){
 g_rand <- sample_gnm(n_nodes, n_edges)
 # Assigning random weight based on real-world weights/freq
 E(g_rand)$weight <- sample(weights, ecount(g_rand), replace = TRUE)
 A_rand <- as_adjacency_matrix(g_rand, sparse = FALSE, attr = "weight")
 C_rand[i] <- ClustBCG(A_rand,"undirected", isolates = "NaN")$GlobalCC
 L_rand[i] <- mean_distance(g_rand, weights = 1/E(g_rand)$weight)
}

# Calculate mean values for null model:
C_rand_mean <- mean(C_rand, na.rm=TRUE)
L_rand_mean <- mean(L_rand, na.rm=TRUE)

# Calculate small_world index:
sigma <- (C_obs/C_rand_mean)/(L_obs/L_rand_mean)

# Calculate p_values:
z_L <- (L_obs - L_rand_mean)/sd(L_rand)
2*pnorm(-(abs(z_L)))

z_C <- (C_obs - C_rand_mean)/sd(C_rand)
2*pnorm(-(abs(z_C)))


# Plots:
par(mfrow=c(1,2))
hist(C_rand, col="skyblue",main="", xlab="Clustering coefficient", xlim=c(0.2, 0.6), ylim=c(0,25))
abline(v=C_obs, col="red", lwd=2)
text(x = par("usr")[1] + 0.1 * diff(par("usr")[1:2]),
   y = par("usr")[4] -0.05 * diff(par("usr")[3:4]),
```

```r
    labels = "a)", font = 1)


hist(L_rand, col = "skyblue",main="", xlab = "Average path length")
abline(v = L_obs, col = "red", lwd = 2)
text(x = par("usr")[1] + 0.1 * diff(par("usr")[1:2]),
    y = par("usr")[4] -0.05 * diff(par("usr")[3:4]),
    labels = "b)", font = 1)

#---------------------------------------------------------#
#           Node statistics for reporting            #
#---------------------------------------------------------#
# Observed local weighted clustering coefficients as defined by Barrat et al (2004):
# Alain Barrat, Marc Barthelemy, Romualdo Pastor-Satorras, Alessandro Vespignani:
# The architecture of complex weighted networks, Proc. Natl. Acad. Sci. USA 101, 3747 (2004)

C_local_obs <- transitivity(g_simple, type = "weighted", weights = weights)
strength <- strength(g_simple, weights=weights)     # node strength
annd <- igraph::knn(g_simple, weights=weights)$knn
anns_w <- as.numeric((A %*% strength) / strength)   # weighted ANNS
node_degree <- degree(g_simple)
node_btw <- betweenness(g_simple, directed = FALSE, weights = 1/weights)
node_closeness <- closeness(g_simple, weights = 1/weights)

w_statistics <- data.frame(
  node = V(g_simple)$name,
  degree = node_degree,
  strength = strength,
  btwness = node_btw,
  closeness = node_closeness,
  cc = C_local_obs,
```

```
  annd = annd,
  anns = anns_w,
  check.names = TRUE
)

# write.csv(w_statistics, "weighted_cc.csv", row.names = TRUE)

# Detecting Louvain communities in weighted network:

louvain_comm <- cluster_louvain(g_simple, weights = E(g_simple)$freq)
membership(louvain_comm) %>% sort()

# Modularity:
Q <- modularity(louvain_comm)

Qs <- replicate(1000, {
  g0 <- g_cleaned
  E(g0)$w <- sample(E(g_cleaned)$freq)
  modularity(cluster_louvain(g0, weights = E(g0)$w))
})
z <- (Q - mean(Qs)) / sd(Qs)
p_two_norm <- 2*pnorm(-abs(z)) # 0.094

V(g_cleaned)$community <- membership(louvain_comm)
num_communities <- length(unique(V(g_cleaned)$community))
community_colors <- brewer.pal(min(num_communities, num_communities), "Set2")

ggraph(g_cleaned, layout = "manual", x = islands_adj$lon_adj, y = islands_adj$lat_adj) +
  geom_edge_arc(aes(edge_width = freq, edge_colour = freq),  strength = 0.7, alpha = 0.8) +
  scale_edge_width(range = c(0.5, 5) , name = "Connection frequency") +
  scale_edge_colour_gradient(low = "grey", high = "black", name = "Connection frequency") +
```

```
  geom_node_point(aes(size = strength, color = as.factor(community)), show.legend = T) +
  scale_size(range=c(3, 15), name = "Node strength", guide = guide_legend(order = 1)) +
  scale_color_manual(values = community_colors, name = "Community")+
  geom_node_text(aes(label = name), repel=TRUE, vjust = -1.2, size = 5) +
  scale_x_reverse() +
  coord_fixed() +
  theme_void() +
  theme(legend.position = "right",
     legend.title = element_text(hjust=0.2)) +
  guides(color = guide_legend(override.aes = list(size = 4)))



#===== Calculate vulnerability metrics per community =====#

#  Assign community to each edge (source and target)

E(g_cleaned)$from_comm <- V(g_cleaned)[ends(g_cleaned, es = E(g_cleaned))[,1]]$community
E(g_cleaned)$to_comm   <- V(g_cleaned)[ends(g_cleaned, es = E(g_cleaned))[,2]]$community
E(g_cleaned)$is_bridge <- E(g_cleaned)$from_comm != E(g_cleaned)$to_comm

# Initialise data frame for community metrics

comm_ids <- sort(unique(V(g_cleaned)$community))

community_metrics <- data.frame(
  community = comm_ids,
  size = NA,                 # Number of nodes
# internal_density = NA,       # Intra-community density
  num_bridges = NA,          # Number of bridging edges
  total_incoming_weight = NA,   # Sum of edge weights from outside
```

```
  avg_degree = NA,           # Mean node degree
  avg_betweenness = NA       # Mean node betweenness
)


for (i in comm_ids) {
  nodes_in_comm <- V(g_cleaned)[community == i]
  subg <- induced_subgraph(g_cleaned, nodes_in_comm)

  # Size of community
  community_metrics$size[i] <- vcount(subg)

  # Internal edge density: actual / possible # Doesn't work with multigraph
  # community_metrics$internal_density[i] <- edge_density(subg, loops = FALSE)%>% round(1)

  # Number of bridging edges (edges that connect to other communities)
  comm_edges <- E(g_cleaned)[from_comm == i | to_comm == i]
  bridges <- comm_edges[comm_edges$is_bridge]
  community_metrics$num_bridges[i] <- length(bridges) %>% round(1)

  # Total incoming weight from other communities
  incoming_weight <- sum(E(g_cleaned)[to_comm == i & is_bridge]$freq)
  community_metrics$total_incoming_weight[i] <- incoming_weight %>% round(1)

  # Node-level stats
  str <- strength(subg, weights = E(subg)$freq)
  betw <- betweenness(subg, weights = 1/E(subg)$freq)
  community_metrics$avg_str[i] <- mean(str) %>% round(1)
  community_metrics$avg_betweenness[i] <- mean(betw) %>% round(1)
}
```

```
# write.csv(community_metrics, "Community vulnerability metrics.csv")


#===================================================#
#            Directed graph                #
#===================================================#

g_directed <- graph_from_data_frame(d = edges, vertices = islands_adj, directed = TRUE)

# Remove edges with freq = 0
drop_idx <- which(E(g_directed)$freq == 0)
g_cleaned_directed <- delete_edges(g_directed, drop_idx)

W <- as_adjacency_matrix(g_cleaned_directed, attr="freq", sparse = FALSE)
R <- pmin(W, t(W)) # Reciprocated weight on each ordered pair

total_trips <- sum(W) # 12,433
recip_trips <- sum(R) # 6,880
non_recip_trips <- total_trips - recip_trips # 5,553

recip_share <- recip_trips/total_trips # 0.5534
reciprocity(g_cleaned_directed, mode = "ratio") # 0.506

###   Average shortest path length   ###
#---------------------------------------

E(g_cleaned_directed)$cost <- 1/(E(g_cleaned_directed)$freq + 1e-4)

D_out <- distances(g_cleaned_directed, mode = "out", weights = E(g_cleaned_directed)$cost)
APL_out <- mean(D_out[is.finite(D_out) & D_out>0])
```

```
D_in  <- mean_distances(g_cleaned_directed, mode = "in",  weights =
E(g_cleaned_directed)$cost)

APL_in <- mean(D_in[is.finite(D_in) & D_in > 0])


###   Community detection (directed/weighted) & modularity  ###

# ------------------------------------------------------------


# Remove 4 isolated nodes:

g_cleaned_directed_removed_nodes <- delete_vertices(g_cleaned_directed,

                                   V(g_cleaned_directed)[name %in% c("Darwin", "Pinta", "Pinzon",
"Wolf")])

#com <- cluster_infomap(g_cleaned_directed_removed_nodes, e.weights =
E(g_cleaned_directed_removed_nodes)$freq)

#vcount(g_cleaned_directed)



set.seed(5432)

comm <- find_partition(g_cleaned_directed_removed_nodes,

          edge_weights =  E(g_cleaned_directed_removed_nodes)$freq) +1

# +1 to avoid community label of 0


names(comm) <- V(g_cleaned_directed_removed_nodes)$name


comm %>% sort()

Q_directed   <- modularity(g_cleaned_directed_removed_nodes, membership=comm,

                weights = E(g_cleaned_directed_removed_nodes)$freq)



Qs_d <- replicate(1000, {

 g0 <- g_cleaned_directed_removed_nodes

 E(g0)$w <- sample(E(g_cleaned_directed_removed_nodes)$freq)

 memb <- find_partition(g0, edge_weights = E(g0)$w) +1
```

```
  names(memb) <- V(g0)$name

  modularity(g0, membership=memb, weights = E(g0)$w)
})
z <- (Q_directed - mean(Qs_d)) / sd(Qs_d)
p_two_norm <- 2*pnorm(-abs(z))


val <- comm[V(g_cleaned_directed)$name]
val[is.na(val)] <- 4:7
V(g_cleaned_directed)$community <- val

V(g_cleaned_directed)$strength <- strength(g_cleaned_directed, mode="all",
                                  weights=E(g_cleaned_directed)$freq)

num_communities2 <- length(unique(V(g_cleaned_directed)$community))
community_colors <- brewer.pal(min(num_communities2, num_communities2), "Set2")


#-------------- Global clustering coefficient -------------#

cc_directed_global_in <- ClustBCG(W, type = "directed")$GlobalinCC
# 0.436

cc_directed_global_out <- ClustBCG(W, type = "directed")$GlobaloutCC
# 0.425

cc_directed_global_cycle <- cc_directed_global_in <- ClustBCG(W, type =
"directed")$GlobalcycleCC
# = cc_directed_global_in
```

```
#------------------ Node-level stats ---------------------#

# Note: Using g_cleaned_directed_removed_nodes for the analysis #

W_node_rm <- as_adjacency_matrix(g_cleaned_directed_removed_nodes,
                         attr = "freq", sparse = FALSE)

node_stats_directed <- data.frame(
  node        = V(g_cleaned_directed_removed_nodes)$name,
  degree_in     = degree(g_cleaned_directed_removed_nodes, mode = "in"),
  degree_out    = degree(g_cleaned_directed_removed_nodes, mode = "out"),
  strength_in   = strength(g_cleaned_directed_removed_nodes, mode = "in",  weights = E(g_cleaned_directed_removed_nodes)$freq),
  strength_out  = strength(g_cleaned_directed_removed_nodes, mode = "out", weights = E(g_cleaned_directed_removed_nodes)$freq),
  betweenness   = betweenness(g_cleaned_directed_removed_nodes, directed = TRUE, weights = E(g_cleaned_directed_removed_nodes)$cost, normalized = TRUE),
  closeness_out = closeness(g_cleaned_directed_removed_nodes,  mode = "out", weights = E(g_cleaned_directed_removed_nodes)$cost, normalized = TRUE),
  closeness_in  = closeness(g_cleaned_directed_removed_nodes,  mode = "in",  weights = E(g_cleaned_directed_removed_nodes)$cost, normalized = TRUE),
  cc_in = ClustBCG(W_node_rm, type = "directed")$inCC,
  cc_out = ClustBCG(W_node_rm, type = "directed")$outCC,
  cc_total = ClustBCG(W_node_rm, type = "directed")$totalCC,
  row.names = NULL
)

write.csv(node_stats_directed, "directed_stats.csv")

# Edge betweenness:
ebtw <- edge_betweenness(g_cleaned_directed_removed_nodes, directed = TRUE, weights = E(g_cleaned_directed_removed_nodes)$cost)
```

```
edges_btw_df <- igraph::as_data_frame(g_cleaned_directed_removed_nodes, what="edges")
%>%
  select(from, to)
edges_btw_df$ebtw <- ebtw



#===== Calculate vulnerability metrics per community for directed network =====#

#  Assign community to each edge (source and target)

E(g_cleaned_directed)$from_comm <- V(g_cleaned_directed)[ends(g_cleaned_directed, es =
E(g_cleaned_directed))[,1]]$community
E(g_cleaned_directed)$to_comm   <- V(g_cleaned_directed)[ends(g_cleaned_directed, es =
E(g_cleaned_directed))[,2]]$community
E(g_cleaned_directed)$is_bridge <- E(g_cleaned_directed)$from_comm !=
E(g_cleaned_directed)$to_comm

# Initialise data frame for community metrics

comm_ids <- sort(unique(V(g_cleaned_directed)$community))

community_metrics_directed <- data.frame(
  community = comm_ids,
  size = NA,                # Number of nodes
  # internal_density = NA,     # Intra-community density
  num_bridges = NA,           # Number of bridging edges
  internal_flow = NA,
  total_inflow = NA,          # Sum of edge weights from outside
  total_outflow = NA,
  closeness_in = NA,
  closeness_out = NA,
  avg_betweenness = NA        # Mean node betweenness
```

```
)

for (i in comm_ids) {
 nodes_in_comm <- V(g_cleaned_directed)[community == i]
 subg <- induced_subgraph(g_cleaned_directed, nodes_in_comm)

 # Size of community
 community_metrics_directed$size[i] <- vcount(subg)

 # Internal edge density: actual / possible # Doesn't work with multigraph
 # community_metrics$internal_density[i] <- edge_density(subg, loops = FALSE)%>% round(1)

 # Number of bridging edges (edges that connect to other communities)
 comm_edges <- E(g_cleaned_directed)[from_comm == i | to_comm == i]
 bridges <- comm_edges[comm_edges$is_bridge]
 community_metrics_directed$num_bridges[i] <- length(bridges) %>% round(2)

 # Total incoming weight from other communities
 incoming_weight <- sum(E(g_cleaned_directed)[to_comm == i & is_bridge]$freq)
 community_metrics_directed$total_inflow[i] <- incoming_weight %>% round(2)

 # Total outgoing weight to other communities
 outgoing_weight <- sum(E(g_cleaned_directed)[from_comm == i & is_bridge]$freq)
 community_metrics_directed$total_outflow[i] <- outgoing_weight %>% round(2)

 # Total internal weight within communities
 internal_weight <- sum(E(g_cleaned_directed)[from_comm == i & !is_bridge]$freq)
 community_metrics_directed$internal_flow[i] <- internal_weight %>% round(2)
```

```
 # Node-level stats
 betw <- betweenness(subg, directed = TRUE, weights = E(subg)$cost)
 community_metrics_directed$avg_betweenness[i] <- mean(betw, na.rm=TRUE) %>% round(2)

 cl_in <- closeness(subg, mode="in", weights= E(subg)$cost, normalized = TRUE)
 community_metrics_directed$closeness_in[i]<- mean(cl_in, na.rm=TRUE) %>% round(2)

 cl_out <- closeness(subg, mode="out", weights= E(subg)$cost, normalized = TRUE)
 community_metrics_directed$closeness_out[i] <- mean(cl_out, na.rm=TRUE) %>% round(2)
 }

# write.csv(community_metrics_directed, "Directed community vulnerability metrics.csv")

#
==========================================================================
========= #
# Robustness test - based on Fortunato and Hric (2016)-
https://doi.org/10.1016/j.physrep.2009.11.002



# Parameters:
sigma  <- 0.5
n_runs <- 1000

g <- g_cleaned_directed_removed_nodes

# ensure weights exist
E(g)$weight0 <- E(g)$freq

islands <- V(g)$name
same_cluster <- matrix(NA_integer_, nrow = n_runs, ncol = vcount(g),
                dimnames = list(paste0("run_", 1:n_runs), islands))
```

```
set.seed(123)

for (k in 1:n_runs) {

  g_pert <- g
  w <- E(g)$weight0

  eps <- runif(length(w), -sigma, sigma)
  E(g_pert)$weight <- w * (1 + eps)

  # community detection (YOUR function)
  part <- find_partition(g_pert, edge_weights = E(g_pert)$weight)



  mem <- part



  # If mem is named, reorder it to match V(g)$name
  if (!is.null(names(mem))) mem <- mem[islands]

  same_cluster[k, ] <- as.integer(mem)
}

# same_cluster is now runs x islands (1000 x 19) with community labels

n_runs <- nrow(same_cluster)
n_islands <- ncol(same_cluster)

co_count <- matrix(0, n_islands, n_islands, dimnames = list(colnames(same_cluster),
                                                            colnames(same_cluster)))
```

```
for (r in 1:n_runs) {
  co_count <- co_count + outer(same_cluster[r, ], same_cluster[r, ], `==`)
}

co_pct <- 100 * co_count / n_runs
vals <- co_pct[upper.tri(co_pct)]

mean(vals)

# 29% stable: weak communities

#=============================================#
#           Plots                    #
#=============================================#

ggraph(g_cleaned_directed, layout = "manual", x = islands_adj$lon_adj, y = islands_adj$lat_adj)
+
  geom_edge_arc(aes(edge_width = freq, edge_colour = freq), strength = 0.7, alpha = 0.8) +
  scale_edge_width(range = c(0.5, 4) , name = "Connection frequency") +
  scale_edge_colour_gradient(low = "grey", high = "black", name = "Connection frequency") +
  geom_node_point(aes(size = strength, color = as.factor(community)), show.legend = T) +
  scale_size(range=c(3, 15), name = "Node strength", guide = guide_legend(order = 1)) +
  scale_color_manual(values = community_colors, name = "Community")+
  geom_node_text(aes(label = name), repel=TRUE, vjust = -1.2, size = 5) +
  scale_x_reverse() +
  coord_fixed() +
  theme_void() +
  theme(legend.position = "right",
      legend.title = element_text(hjust=0.2))+
  guides(color = guide_legend(override.aes = list(size = 4)))
```

```
# Detect strong bridges (i.e., Edges if removed increase the number of strongly connected components)

strong_bridges <- function(g) {
  base <- components(g, mode = "strong")$no
  sapply(seq_len(gsize(g)), function(eid) {
    components(delete_edges(g, eid), mode = "strong")$no > base
  })
}
components(g_cleaned_directed_removed_nodes, mode="strong")

#============================================#
#       Composite weight with SST        #
#============================================#
library(dplyr)
library(tidyverse)

# Pivot similarity df into longer df:
SST_sim_df <- edges %>%
  select(freq, contains("sim_")) %>%
  filter(freq>0) %>%
  pivot_longer(cols = starts_with("sim"),
               names_to = "sim_month",
               values_to = "similarity")

mon_abbr <- sub("^[^_]*_", "", SST_sim_df$sim_month) %>% toupper()
month_order <- c("FEB", "MAR", "APR", "MAY", "JUN", "JUL",
                 "AUG", "SEP", "OCT", "NOV", "DEC", "JAN_16")
SST_sim_df$month <- mon_abbr %>%
  factor(levels = month_order)
```

```
fit <- aov(similarity ~ month, data = SST_sim_df)
# QQ plot
qqnorm(residuals(fit)); qqline(residuals(fit))
hist(residuals(fit), breaks = "FD")
# Non-normal residuals

# Kruskall-Wallis test
kruskal.test(similarity ~ month, data = SST_sim_df)
# Kruskal-Wallis chi-squared = 209.34, df = 11, p-value < 2.2e-16


pw <- statsExpressions::pairwise_comparisons(
  data = SST_sim_df,
  x = month, y = similarity,
  type = "nonparametric",
  p.adjust.method = "BH"
) %>%
  mutate(groups = purrr::pmap(.l = list(group1, group2), .f = c),
         stars = case_when(
           p.value <= 0.001 ~ "***",
           p.value <= 0.01  ~ "**",
           p.value <= 0.05  ~ "*",
           TRUE ~ "ns"
         ))


pw <- pw %>%
  filter(!is.na(stars))

p <- SST_sim_df %>% ggbetweenstats(
```

```
  x = month,
  y = similarity,
  type = 'nonparametric',
  pairwise_comparisons = TRUE,
  pairwise.display = "none",
  violin.args = list(width = 0),
  point.args = list(alpha=0, size=0),
  centrality.type = "nonparametric",
  centrality.point.args = list(size = 2, color="black"),
  centrality.label.args = list(nudge_y=0.8, segment.linetype=3, nudge_x=0)
)
p
y_position = c(2, rep(0, 43))
for (i in 2:length(y_position)) {
  y_position[i] = y_position[i-1] + 0.1
}

p + ggsignif::geom_signif(
  comparisons   = pw$groups,
  map_signif_level = TRUE,
  test = NULL,
  annotations   = pw$stars,
  y_position    = y_position,
  tip_length    = 0.01,
  textsize      = 4,
  vjust = 0.85
) +
  xlab("Month") + ylab("SST similarity")+
  theme_classic() +
  theme(legend.position = "none")
```

```
#=================================================#
#     Network with Composite weight for April      #
#=================================================#
g_april = g_cleaned_directed


E(g_april)$cost <- 1/(E(g_april)$freq*E(g_april)$sim_apr + 1e-4)


E(g_april)$weights <- E(g_april)$freq*E(g_april)$sim_apr


D_out_apr <- distances(g_april, mode = "out", weights = E(g_april)$cost)
APL_out_apr <- mean(D_out_apr[is.finite(D_out_apr) & D_out_apr>0])


D_in_apr  <- distances(g_april, mode = "in",  weights = E(g_april)$cost)
APL_in_apr <- mean(D_in_apr[is.finite(D_in_apr) & D_in_apr > 0])


###   Community detection (directed/weighted) & modularity  ###
# ------------------------------------------------------------


# Remove 4 isolated nodes:
g_april_removed_nodes <- delete_vertices(g_april,
                                 V(g_april)[name %in% c("Darwin", "Pinta", "Pinzon", "Wolf")])
#com <- cluster_infomap(g_cleaned_directed_removed_nodes, e.weights =
E(g_cleaned_directed_removed_nodes)$freq)
#vcount(g_cleaned_directed)



set.seed(1234)
comm_apr <- find_partition(g_april_removed_nodes,
              edge_weights =  E(g_april_removed_nodes)$weights, resolution =1, niter=2) +1
# +1 to avoid community label of 0
names(comm_apr) <- V(g_april_removed_nodes)$name
```

```
comm_apr %>% sort()
Q_directed_apr   <- modularity(g_april_removed_nodes, membership=comm_apr,
                    weights = E(g_april_removed_nodes)$weights)


Qs_d_apr <- replicate(1000, {
  g0 <- g_april_removed_nodes
  E(g0)$w <- sample(E(g_april_removed_nodes)$weights)
  memb <- find_partition(g0, edge_weights = E(g0)$w, resolution =1, niter=2) +1
  names(memb) <- V(g0)$name

  modularity(g0, membership=memb, weights = E(g0)$w)
})
z <- (Q_directed_apr - mean(Qs_d_apr)) / sd(Qs_d_apr)
# 1.93
# p-value
2*pnorm(-abs(z)) # 0.053 borderline significant


val_apr <- comm_apr[V(g_april)$name] # 4 communities
val_apr[is.na(val_apr)] <- 5:8
V(g_april)$community <- val_apr

V(g_april)$strength <- strength(g_april, mode="all",
                                weights=E(g_april)$weights)

#-------------- Global clustering coefficient -------------#

W_apr <- as_adjacency_matrix(g_april,attr = "weights", sparse = FALSE )
```

```
cc_directed_global_in_apr <- ClustBCG(W_apr, type = "directed")$GlobalinCC
# 0.446

cc_directed_global_out_apr <- ClustBCG(W_apr, type = "directed")$GlobaloutCC
# 0.415

cc_directed_global_cycle_apr <- ClustBCG(W_apr, type = "directed")$GlobalcycleCC
# = cc_directed_global_in



#------------------ Node-level stats ---------------------#
# Note: Using g_april_removed_nodes for the analysis #

W_node_rm_apr <- as_adjacency_matrix(g_april_removed_nodes,
                    attr = "weights", sparse = FALSE)

node_stats_directed_apr <- data.frame(
  node        = V(g_april_removed_nodes)$name,
  degree_in     = degree(g_april_removed_nodes, mode = "in"),
  degree_out    = degree(g_april_removed_nodes, mode = "out"),
  strength_in   = strength(g_april_removed_nodes, mode = "in",  weights =
E(g_april_removed_nodes)$weights),
  strength_out  = strength(g_april_removed_nodes, mode = "out", weights =
E(g_april_removed_nodes)$weights),
  betweenness   = betweenness(g_april_removed_nodes, directed = TRUE, weights =
E(g_april_removed_nodes)$cost, normalized = TRUE),
  closeness_out = closeness(g_april_removed_nodes,  mode = "out", weights =
E(g_april_removed_nodes)$cost, normalized = TRUE),
  closeness_in  = closeness(g_april_removed_nodes,  mode = "in",  weights =
E(g_april_removed_nodes)$cost, normalized = TRUE),
  cc_in = ClustBCG(W_node_rm_apr, type = "directed")$inCC,
  cc_out = ClustBCG(W_node_rm_apr, type = "directed")$outCC,
  cc_total = ClustBCG(W_node_rm_apr, type = "directed")$totalCC,
```

```
  row.names = NULL
)

# write.csv(node_stats_directed_apr, "April_directed_stats.csv")

# Edge betweenness:
ebtw_apr <- edge_betweenness(g_april_removed_nodes, directed = TRUE, weights =
E(g_april_removed_nodes)$cost)

edges_btw_df_apr <- igraph::as_data_frame(g_april_removed_nodes, what="edges") %>%
  select(from, to)
edges_btw_df_apr$ebtw <- ebtw_apr

edges_btw_df_apr %>% arrange(desc(ebtw))

#===== Calculate vulnerability metrics per community for directed network =====#

#  Assign community to each edge (source and target)

E(g_april)$from_comm <- V(g_april)[ends(g_april, es = E(g_april))[,1]]$community
E(g_april)$to_comm   <- V(g_april)[ends(g_april, es = E(g_april))[,2]]$community
E(g_april)$is_bridge <- E(g_april)$from_comm != E(g_april)$to_comm

# Initialise data frame for community metrics

comm_ids_apr <- sort(unique(V(g_april)$community))

community_metrics_directed_apr <- data.frame(
  community = comm_ids_apr,
  size = NA,                # Number of nodes
  # internal_density = NA,     # Intra-community density
```

```r
  num_bridges = NA,          # Number of bridging edges
  internal_flow = NA,
  total_inflow = NA,          # Sum of edge weights from outside
  total_outflow = NA,
  closeness_in = NA,
  closeness_out = NA,
  avg_betweenness = NA        # Mean node betweenness
)


for (i in comm_ids_apr) {
  nodes_in_comm <- V(g_april)[community == i]
  subg <- induced_subgraph(g_april, nodes_in_comm)

  # Size of community
  community_metrics_directed_apr$size[i] <- vcount(subg)

  # Number of bridging edges (edges that connect to other communities)
  comm_edges <- E(g_april)[from_comm == i | to_comm == i]
  bridges <- comm_edges[comm_edges$is_bridge]
  community_metrics_directed_apr$num_bridges[i] <- length(bridges) %>% round(2)

  # Total incoming weight from other communities
  incoming_weight <- sum(E(g_april)[to_comm == i & is_bridge]$weights)
  community_metrics_directed_apr$total_inflow[i] <- incoming_weight %>% round(2)

  # Total outgoing weight to other communities
  outgoing_weight <- sum(E(g_april)[from_comm == i & is_bridge]$weights)
  community_metrics_directed_apr$total_outflow[i] <- outgoing_weight %>% round(2)

  # Total internal weight within communities
```

```
  internal_weight <- sum(E(g_april)[from_comm == i & !is_bridge]$weights)
  community_metrics_directed_apr$internal_flow[i] <- internal_weight %>% round(2)


  # Node-level stats
  betw <- betweenness(subg, directed = TRUE, weights = E(subg)$cost)
  community_metrics_directed_apr$avg_betweenness[i] <- mean(betw, na.rm=TRUE) %>%
round(2)

  cl_in <- closeness(subg, mode="in", weights= E(subg)$cost, normalized = TRUE)
  community_metrics_directed_apr$closeness_in[i]<- mean(cl_in, na.rm=TRUE) %>% round(2)

  cl_out <- closeness(subg, mode="out", weights= E(subg)$cost, normalized = TRUE)
  community_metrics_directed_apr$closeness_out[i] <- mean(cl_out, na.rm=TRUE) %>%
round(2)

}

#write.csv(community_metrics_directed_apr, "Directed community vulnerability metrics -
April.csv")


#==============================================#
#           Plots                    #
#==============================================#
num_communities2_apr <- length(unique(V(g_april)$community))
community_colors_apr <- brewer.pal(min(num_communities2_apr, num_communities2_apr),
"Dark2")

ggraph(g_april, layout = "manual", x = islands_adj$lon_adj, y = islands_adj$lat_adj) +
  geom_edge_arc(aes(edge_width = freq, edge_colour = sim_apr), strength = 0.7, alpha = 0.8) +
  scale_edge_width(range = c(0.5, 4) , name = "Connection frequency") +
```

```
 scale_edge_colour_gradient2(low = "darkblue",midpoint= 0.6, mid = "lightblue", high =
"darkred", name = "SST similarity") +

 geom_node_point(aes(size = strength, color = as.factor(community)), show.legend = T) +

 scale_size(range=c(3, 15), name = "Node strength", guide = guide_legend(order = 1)) +

 scale_color_manual(values = community_colors_apr, name = "Community")+

 geom_node_text(aes(label = name), repel=TRUE, vjust = -1.2, size = 5) +

 scale_x_reverse() +

 coord_fixed() +

 theme_void() +

 theme(legend.position = "right",

    legend.title = element_text(hjust=0.2))+

 guides(color = guide_legend(override.aes = list(size = 4)))
```
